# Visible‐Light Mediated Activation of 1,3‐Dienes and Allenes via Energy Transfers

**DOI:** 10.1002/chem.202502499

**Published:** 2025-10-27

**Authors:** Scarica Gabriele, Chiminelli Maurizio, Corrieri Matteo, Cerveri Alessandro, Maggi Raimondo, Maestri Giovanni, Lanzi Matteo

**Affiliations:** ^1^ Department of Chemistry Life Sciences and Environmental Sustainability Università di Parma Parco Area delle Scienze 17/A Parma 43124 Italy

**Keywords:** 1,3‐dienes, allenes, energy transfer, photocatalysis

## Abstract

Nature has inspired chemists toward the development of photocatalytic processes. By harvesting visible light, chemists can access alternative reaction pathways that bypass traditional thermal methods. Energy transfer (EnT) stands out for enabling the activation of otherwise inert substrates, generating the corresponding triplet states. This review highlights and compare the recent advances in EnT‐mediated activation of conjugated 1,3‐dienes and allenes (cumulated 1,2‐dienes), which represent an underdeveloped avenue for synthetic purposes. These approaches enable novel transformations, ranging from photoisomerization to challenging cycloaddition. These findings can have broad implications across synthetic chemistry and materials science, offering energy‐efficient and atom‐economical routes to complex molecular architectures. Studies employing indirect functionalization, in which these motifs are not directly activated via EnT, are explicitly excluded from this review.

## Graphical abstract

Nature has inspired chemists to develop photocatalytic processes that harness visible light, offering alternative reaction pathways beyond traditional thermal methods. Energy Transfer (EnT) is a particularly promising strategy, enabling transformations such as isomerization, deracemization, cycloadditions, and cascade reactions. EnT can activate otherwise inert substrates, providing energy‐efficient and atom‐economical routes to complex molecular architectures. This review focuses on recent advances in EnT‐mediated activation of conjugated 1,3‐dienes and allenes, highlighting their synthetic potential and applications in material science.

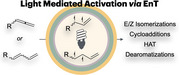

## 1 Introduction

Chemists have drawn profound inspiration from nature, particularly in the development of photochemistry. Beginning with the pioneering work of Ciamician, which laid the foundation for much of modern photochemistry, the natural process of photosynthesis has driven generations of chemists to explore light‐mediated chemical transformations.^[^ ^1^, ^2^, ^3^, ^4^ ^]^

Harvesting visible light has uncovered entirely new and complementary reaction pathways offering alternatives to traditional processes that rely purely on thermal energy.^[^ ^5^, ^6^ ^]^ This allowed researchers to move beyond conventional heat‐driven chemistry and embrace more sustainable and energy‐efficient approaches.

Photocatalysis has emerged as a cornerstone of light‐mediated chemical reactions and played a pivotal role in the advancement of the field.^[^ ^7^, ^8^, ^9^, ^10^ ^]^ Photocatalysts, substances capable of absorbing light and transferring energy to non absorbing substrates, enable chemists to orchestrate intricate reactions with unparalleled efficiency and selectivity.^[^ ^11^, ^12^ ^]^ The ability to induce chemical reactivity in molecules that would otherwise remain inert has made photocatalysis an indispensable tool in modern organic chemistry.^[^ ^13^, ^14^, ^15^ ^]^

**Figure 1:**
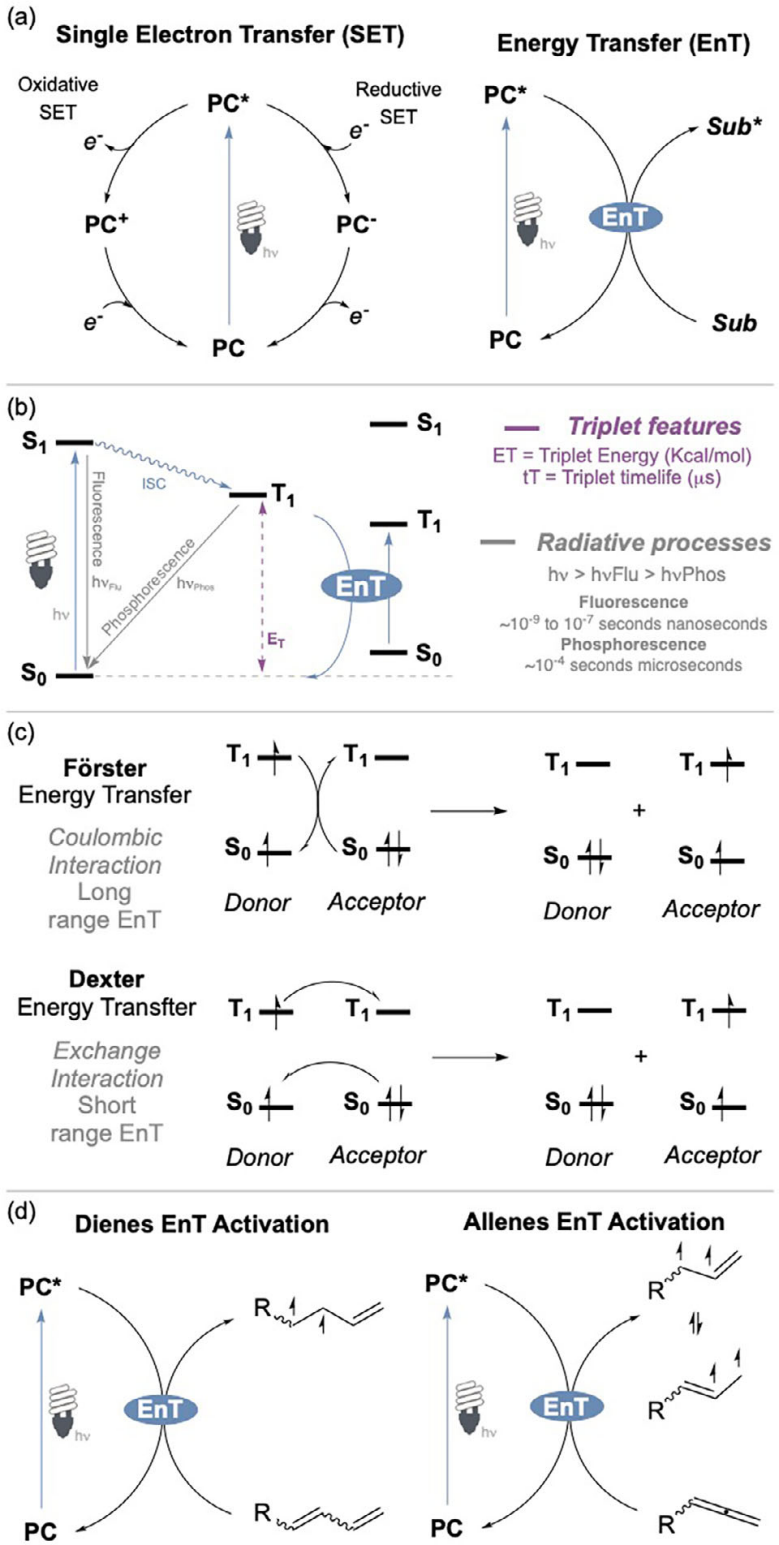
a) Photocatalytic mechanisms; b) Jablonski diagram illustrating energy transfer (EnT) activation of substrates; c) EnT mechanism; d) EnT activation of 1,3‐dienes and allenes.

The energy transfer (EnT) process has proven to be a highly valuable tool in photocatalysis, allowing access to energetic triplet states that can overcome otherwise insurmountable energy barriers (Figure 1a).^[^ ^16^, ^17^, ^18^ ^]^ The absorption of an energetic photon, typically in the blue or purple region of the visible‐light spectrum, by a photocatalyst populates the singlet excited state of the latter (Figure 1b). The sequent intersystem crossing (ISC) permits the state transition into an excited triplet state.^[^ ^19^ ^]^ The triplet state **T_1_** is characterized by a significantly enhanced lifetime compared to **S_1_**, ranging from nanoseconds to microseconds. This feature is essential for an efficient bimolecular quenching event to occur. Although radiative processes can occur from both the **T_1_** and the **S_1_** states, the former could also undergo an EnT to a ground‐state acceptor, commonly referred to as the substrate.^[^ ^20^ ^]^

From a molecular perspective, we can categorize three distinct types of EnT mechanisms: primitive EnT via an emission‐absorption process; the Förster resonance EnT; and the Dexter EnT (Figure 1c).^[^ ^21^, ^22^, ^23^, ^24^ ^]^ Primitive EnT occurs through a simple emission‐absorption process between two species with suitably overlapping spectral bands. The Förster resonance EnT operates via a nonradiative mechanism, relying on the close spatial proximity between the donor and acceptor molecules. Dexter EnT mechanism, in contrast, involves a more intricate two‐electron exchange process, in which the donor transfers an electron from its singly occupied molecular orbital (SOMO) to the lowest unoccupied molecular orbital (LUMO) of the acceptor, while simultaneously receiving an electron from the highest occupied molecular orbital (HOMO) of the acceptor. This exchange facilitates the population of the **T_1_** state of the acceptor while simultaneously regenerating the ground state of the donor.^[^ ^25^ ^]^ At the molecular level, the Dexter EnT mechanism requires a degree of orbital overlap between the donor and the acceptor, implying that they must be in proximity for the process to occur.^[^ ^26^ ^]^

In solution, this proximity is often achieved through the diffusion‐driven formation of a transient, solvent‐shared encounter complex. The Dexter‐type EnT can take place within this supramolecular entity, whose subsequent dissociation liberates the activated acceptor, which can potentially engage in a chemical reaction.^[^ ^27^ ^]^

The EnT is inherently a dual electron transfer process, whose efficiency is governed by a combination of several fundamental factors. Within this theoretical framework, three primary parameters play a crucial role in determining the overall dynamics of the process: (a) The thermodynamic driving force serves as a critical determinant, as it reflects the free energy difference between the initial and final states of the system; (b) The reorganization energy barrier represented by the structural and solvation changes required for the electrons to successfully transfer from the donor to the acceptor; (c) The electronic overlap between the wavefunctions of the donor and the acceptor states is essential for an efficient electron transfer.^[^ ^28^ ^]^

Among the parameters that an EnT‐based protocol could require to optimize, the authors would suggest evaluating the following parameters in advance. The feasibility of EnT events for synthetic applications is often assessed by focusing primarily on the first factor (a). Indeed, the driving force can be initially evaluated by comparing the triplet energies (E_T_) of both the donor and the acceptor. This preliminary assessment can be carried out experimentally by collecting phosphorescence and absorption spectra; alternatively, computational methods that rely on high‐quality quantum‐mechanical models have proved to afford very reliable values at relatively reasonable computational costs; finally, predictive tools can give rapid hints that are often good estimates. Because the efficiency of the EnT depends on the energetic compatibility of the two components, if the triplet energy of the acceptor is higher than that of the donor, the EnT will be either inefficient or outright not possible.

An additional persistent and critical challenge in EnT catalysis is the selection of an appropriate sensitizer.^[^ ^29^ ^]^ The conventional approach involves screening a range of catalysts to optimize reactivity. However, this method can significantly reduce productivity, as it requires extensive experimental trials. To streamline this process, researchers often rely on Stern–Volmer luminescence quenching studies. This analytical technique provides insights into the interaction dynamics between an emitter and a quencher, which is an entity that diminishes the intensity of the luminescence of the emitter by absorbing or dissipating its energy. Beyond luminescence quenching studies, mechanistic investigations, including DFT calculations, can further elucidate the suitability of a sensitizer.^[^ ^30^ ^]^

Finally, EnT reactions are highly sensitive to the number of absorbed photons. Consequently, the irradiation intensity plays a crucial role in determining the reaction efficiency. Several key factors influence light absorption, including the spectral distribution of the light source and an efficient light transfer mechanism. Temperature control is another critical aspect of photochemical reactions since it can affect reaction rates and selectivity. Additionally, reactor dimensions must be considered, as changes in reactor size can influence photon flux and, consequently, the reaction rate. Solvent polarity can induce complementary solvatochromism, affecting reaction outcomes.

Understanding these effects is crucial for designing optimized reaction conditions that maximize photocatalytic efficiency. A major limitation in EnT‐driven catalysis is the necessity of conducting reactions under an oxygen‐free atmosphere. Triplet oxygen, in its ground state, is often a potent quencher thanks to its narrow triplet energy. Therefore, an inert atmosphere, typically argon or nitrogen, is often required.

With this review, we aim to provide the readers with a comprehensive overview of recent studies on the light‐mediated EnT activation of conjugated and cumulated dienes (Figure 1d). Several reviews have covered the EnT activation of alkenes conjugated with auxochromes, such as styrenes and acrylates, which allow for an efficient activation but do not participate in the subsequent reaction. On the contrary, our focus herein is specifically on 1,3‐dienes and allenes, highlighting the unique photochemical transformations that become available, thanks to the presence of two cooperating C─C double bonds.^[^ ^31^, ^32^, ^33^, ^34^, ^35^, ^36^, ^37^, ^38^ ^]^

Processes in which the energy of light is used to excite molecules containing double bonds via EnT cover a large variety of chemical transformations, including isomerizations, several cycloadditions, and radical cascades. Beyond synthetic chemistry, the *cis*/*trans* photochemical isomerization plays a crucial role in vision, in which 11‐*cis*‐retinal converts to all‐*trans*‐retinal upon light absorption.^[^ ^39^, ^40^, ^41^ ^]^ Among cycloadditions, the photochemical alkene‐alkene [2 + 2] cycloaddition that has often been performed using high‐energy UV irradiation is now often realized through a photo‐catalytic approach instead, exploiting less hazardous photons.^[^ ^25^, ^42^ ^]^ Taken together, these transformations provide a basis for advanced applications in materials science, for the synthesis of pharmaceuticals, and for the development of more sustainable chemical methods.^[^ ^13^, ^43^, ^44^ ^]^

The review will now focus on the EnT activation of conjugated 1,3‐dienes and cumulated 1,2‐ones, highlighting the complementary nature of these two functional groups and the unique opportunities that they provide compared to more popular substrates. Studies that involve indirect functionalization, in which these motifs are not directly activated through EnT, are explicitly excluded from this review.

## 2 Activation of Conjugated 1,3‐Dienes

**Scheme 1:**
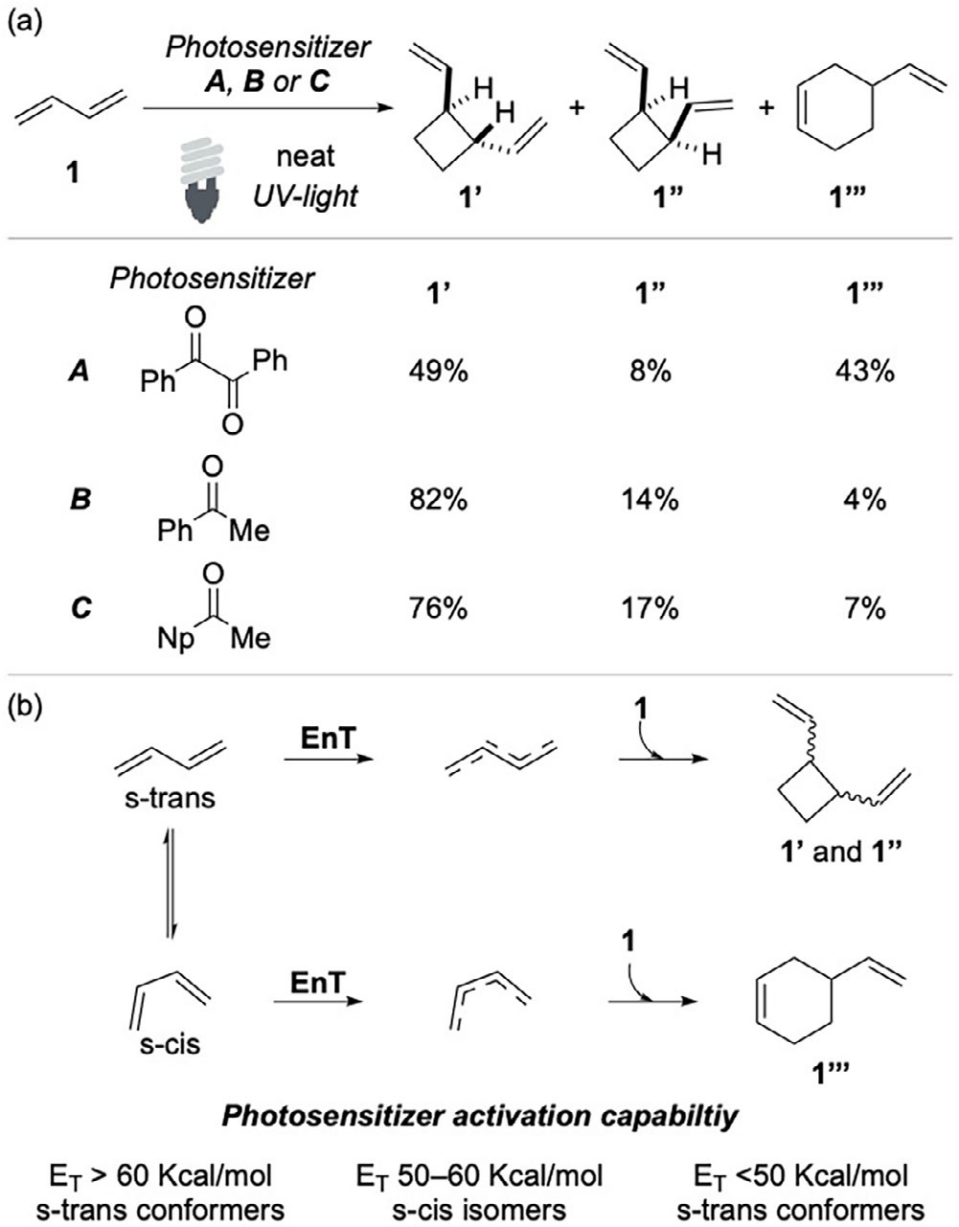
Butadiene cycloaddition upon sensitization.

The photoisomerization of conjugated systems has been extensively explored since the 1950s.^[^ ^45^, ^46^, ^47^ ^]^ Preliminary studies on the dimerization of the simplest 1,3‐diene, butadiene, were reported by Hammond, who studied the behavior of them in the presence of various photosensitizers (Scheme 1a).^[^ ^48^ ^]^ The study revealed that the nature of the latter had a significant influence on the outcome of the photochemical reaction, leading to the formation of a variable mixture of dimeric products. Specifically, both [2 + 2] and [4 + 2] cycloaddition products were observed, with their relative proportions strongly dependent on the specific sensitizer utilized. The study was then extended to cyclopentadiene and isoprene.^[^ ^49^ ^]^

Later on, in 1965, Hammond and coworkers conducted a comprehensive study on the dimerization of photosensitized of isoprene and butadiene, demonstrating that product distribution is strongly influenced by the E_T_ of the sensitizer.^[^ ^50^ ^]^ Upon EnT, dienes are promoted to their triplet excited states, leading to divergent reactivity: *cisoid* triplets favor the formation of cyclohexene derivatives via formal [4 + 2] cycloaddition, whereas *transoid* species predominantly yield cyclobutanes and cyclooctadienes, via formal [2 + 2] and [4 + 4] pathways (Scheme 1b).

Sensitizers with E_T_ > 60 kcal/mol, such as aromatic ketones and aldehydes, or < 50 kcal/mol preferentially activate the *S*‐trans conformer, which is the most abundant ground‐state species. In contrast, sensitizers with an E_T_ in the 50–60 kcal/mol range selectively engage the *S*‐cis isomer.

Kinetic analyses support the dual‐channel mechanism in which both conformers could be activated, providing in turn both the *cisoid*‐ and the *transoid*‐ triplets. Bimolecular rate constants approached the diffusion limit in both cases. Quenching experiments with azulene revealed preferential activation of the *cisoid*‐ triplet, which has a longer lifetime. Collectively, the data supported an EnT‐based activation mode and effectively ruled out alternative addition/elimination mechanisms. Furthermore, these studies highlighted the intricacies of the photochemical behavior of 1,3‐dienes.

**Scheme 2:**
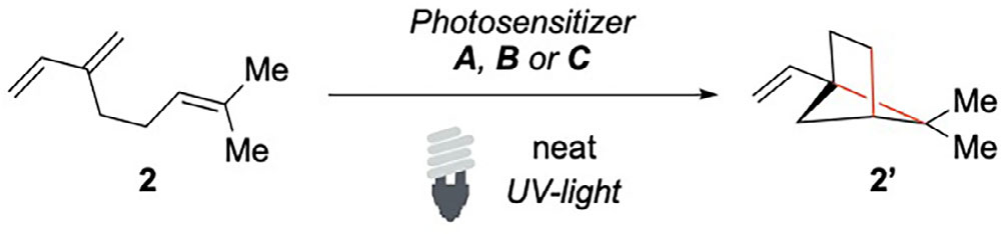
Cross‐cycloaddition of Mircene.

The same group explored the intramolecular cycloaddition of myrcene and structurally related analogues.^[^ ^51^, ^52^ ^]^ Upon photoirradiation in the presence of a sensitizer, such as acetophenone or benzophenone, myrcene **2** and similar terpenes undergo cycloaddition to yield the corresponding bicyclo[2.2.1]hexane derivatives **2′** (Scheme 2).

When both *cis*‐ and *trans*‐3‐methylene‐1,5‐heptadienes were employed, the reaction afforded a mixture of the *endo‐* and the *exo‐* products. Interestingly, the *endo‐/exo‐* ratio of these products was unchanged regardless of the isomeric composition of the starting material, with a preference for the *endo*‐ isomer. This observation points to the formation of a common biradical intermediate in which rapid bond rotation precedes the ring‐closing event. These findings differ significantly from earlier studies, in which the direct irradiation of myrcene in the absence of a photosensitizer predominantly gave a cyclobutene product.

Motivated by these insights, Pan and coworkers more recently investigated the [2 + 2] and [2 + 1] cycloaddition pathways of myrcene as a strategy for the synthesis of novel energetic materials.^[^ ^53^ ^]^ It is worth noting that similar approaches are a promising tool to meet the increasing demand for renewable chemical feedstocks.

**Scheme 3:**
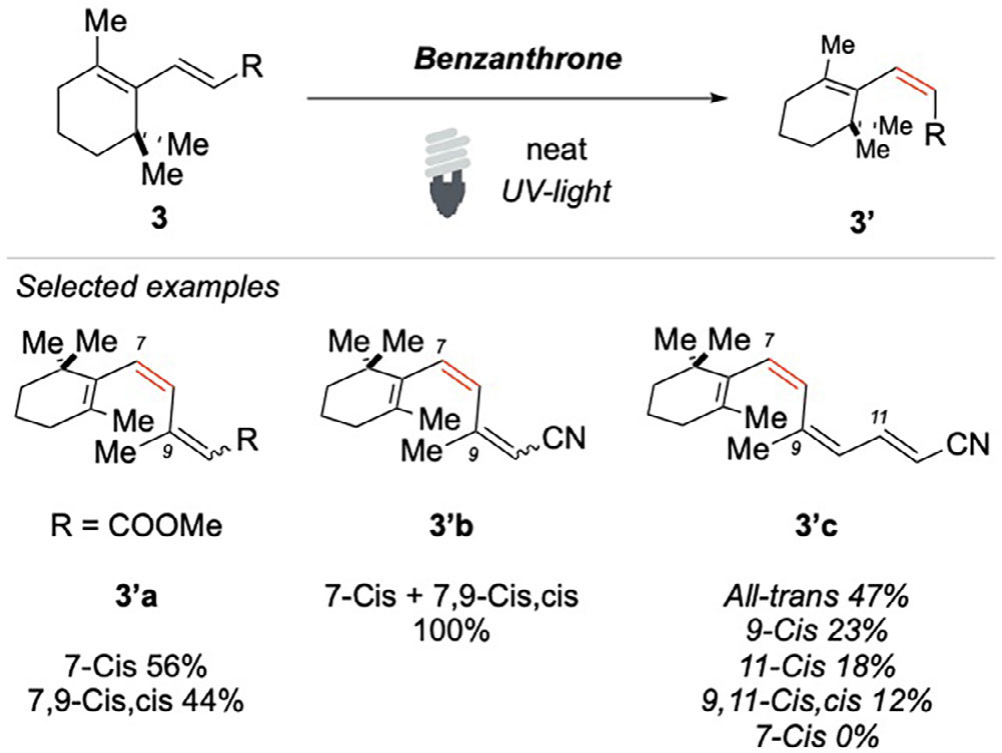
En‐Tbased photo EnTisomerization of Vitamin A derivatives.

In 1976, Hammond and coworkers demonstrated that the sensitization of dienes and trienes in the vitamin A series provides access to highly hindered 7‐*cis* isomers, which are otherwise inaccessible under direct photoirradiation.^[^ ^54^ ^]^ The outcome of the isomerization is dictated by the E_T_ of the sensitizer: high‐energy donors (>74 kcal/mol) indiscriminately activate both isomers, whereas lower‐energy sensitizers enable a selective, unidirectional conversion to the *cis* isomer. This selectivity is attributed to a large (∼15 kcal/mol) energy gap between the twisted *cisoid*‐ and the nearly planar *transoid*‐ triplets, which can be exploited using a suitable EnT donor (Scheme 3).

In contrast, tetraenes and pentaenes fail to yield the corresponding 7‐*cis* isomer. This divergent behavior is explained by a conformational change in the relaxed triplet state: whereas dienes and trienes adopt perpendicular geometries to minimize the spin repulsion from mono‐occupied molecular orbitals that are in relatively proximity, higher polyenes remain planar in the excited state, thanks to a larger degree of spin delocalization. Consequently, the isomerization of tetraenes and pentaenes is governed solely by thermodynamics, inherently disfavoring the sterically encumbered 7‐*cis* configuration. This model is supported by photostationary‐state studies, azulene quenching experiments, and the consistent absence of 7‐*cis* isomers even upon low‐temperature irradiation.

**Scheme 4:**
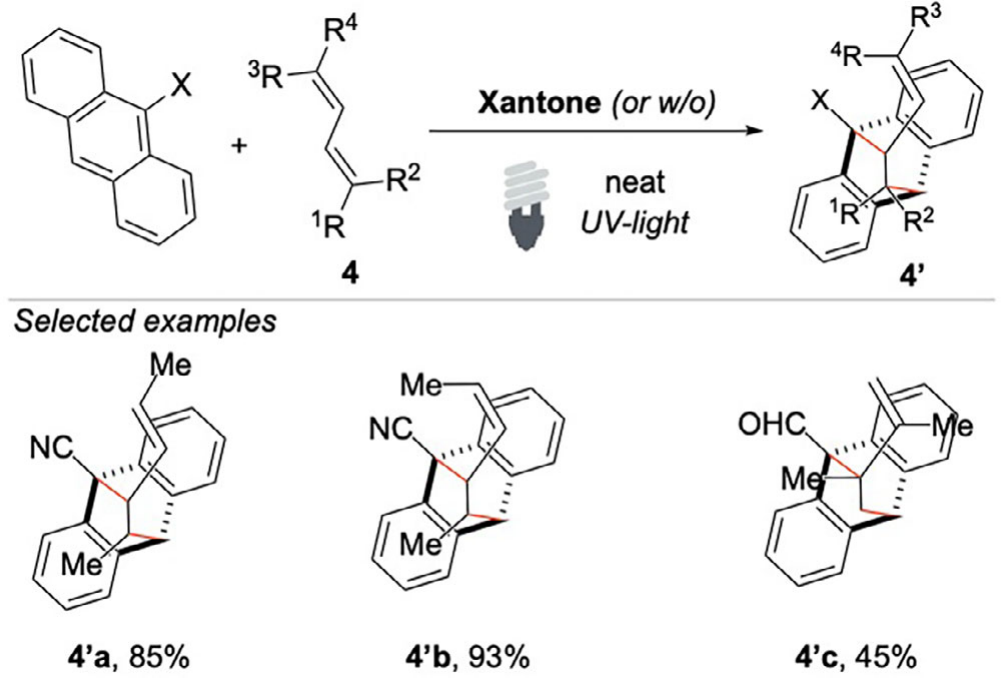
Cycloaddition of acyclic 1,3‐Dienes to anthracene.

The reactivity of 1,3‐dienes with polycyclic aromatic hydrocarbons was also investigated.^[^ ^55^, ^56^, ^57^ ^]^ The study focuses on the stereospecific 1,2‐photocycloaddition of acyclic dienes with 9‐cyanoanthracene and 9‐anthraldehyde, resulting in the formation of 9,10‐ethanoanthracene derivatives (Scheme 4).

The authors proposed that the reaction involves the formation of an exciplex, whose configuration dictates the outcome of the addition by allowing either *cis*‐ or *trans*‐ substituted 1,3‐dienes to react in a stereospecific manner. It is important to note that no 1,4‐adduct was observed in any of these reactions, which would have involved a formal [4 + 4] cycloaddition. These results suggest that the reaction mechanism of photochemical addition could be significantly influenced by the spatial arrangement of the components within the exciplex intermediate. In the present case, the observed formal [4 + 2] photocycloaddition represents a complementary method to the popular Diels–Alder reaction. Notably, the photocatalytic reaction of anthracene with acyclic 1,3‐dienes is most likely a stepwise [4 + 2] photocycloaddition because the corresponding concerted process is symmetry‐forbidden according to the Woodward–Hoffmann rules.

**Scheme 5:**
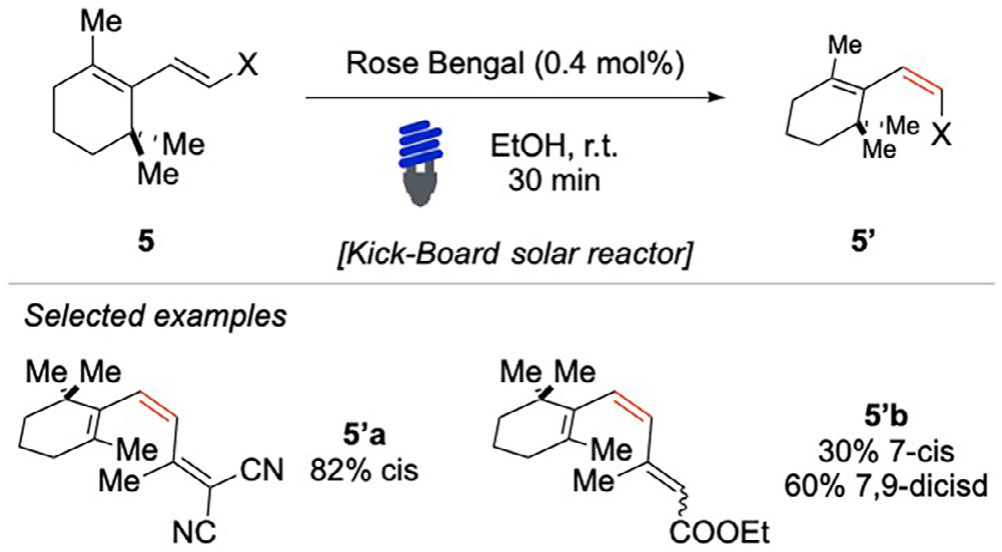
EnT‐based photoisomerization via solar irradiation.

By harnessing the power of natural sunlight, Liu and coworkers have successfully achieved EnT‐based isomerizations of both 1,3‐dienes and trienes through direct solar irradiation (Scheme 5).^[^ ^58^ ^]^

This innovative approach highlights the potential of sustainable photochemistry, eliminating the need for artificial light sources and reducing energy consumption. In their study, they focused on the isomerization of *cis*‐ionone derivatives, employing a Kick–Beard solar reactor specifically designed to optimize exposure to natural sunlight. Using an organic photocatalyst, the isomerization process proceeded with remarkable efficiency, yielding highly isomerized products under mild and eco‐friendly conditions.

**Scheme 6:**
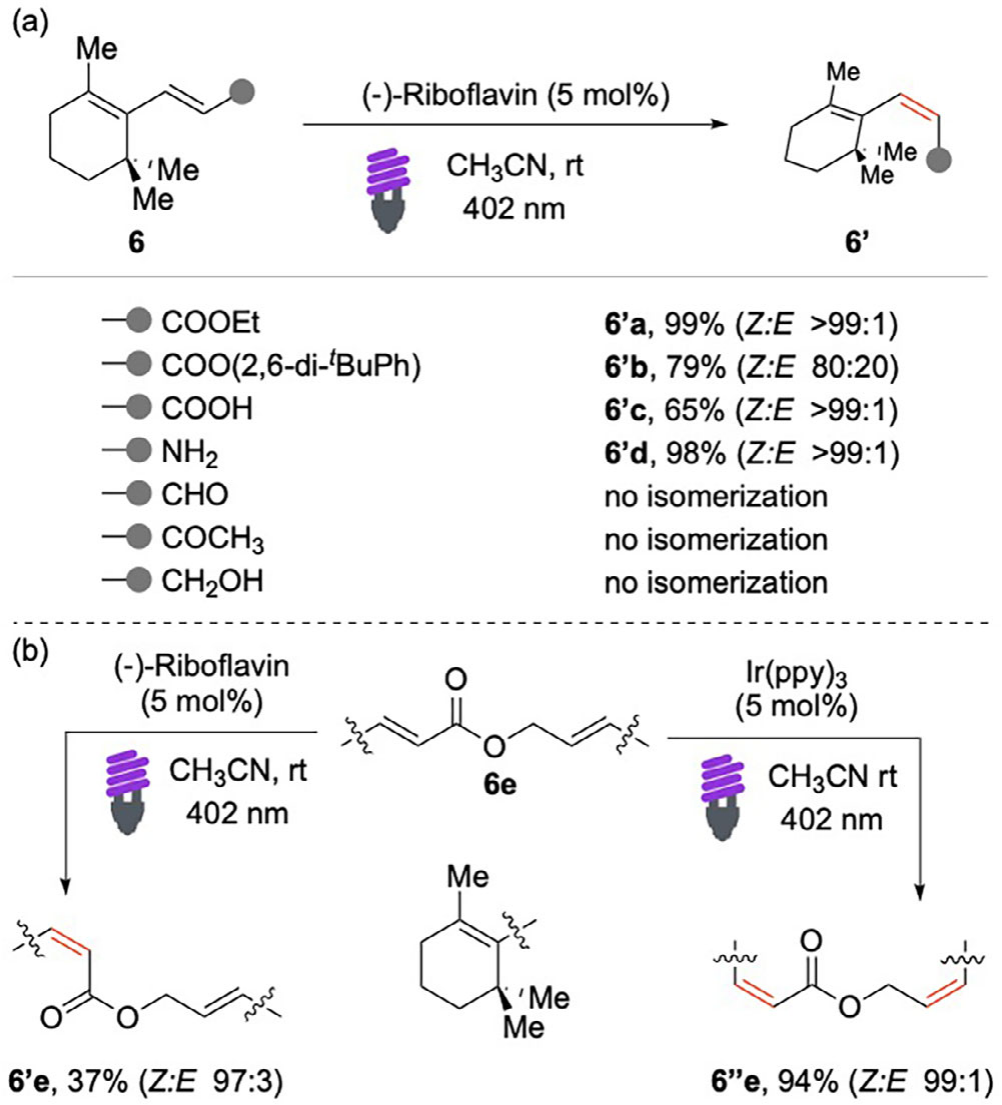
EnT bisomerization of dienes **6**.

Gilmour and colleagues achieved the *cis*/*trans* isomerization of β‐ionyl derivatives **6** using the natural polyene (−)‐riboflavin (E_T_ = 49.9 kcal/mol) as photocatalyst (Scheme 6a).^[^ ^59^ ^]^ The protocol proved to be efficient for esters **6′a**, including bulky ones, as well as for acids **6′c** and amides **6′d**. On the other hand, the lack of reactivity of allylic aldehydes, ketones, and alcohols presents opportunities for chemoselectivity. For instance, when compound **6e** was exposed to visible light irradiation, only the ester portion underwent isomerization, while the alcohol moiety remained untouched (Scheme 6b). This selectivity is attributed to the photochemical properties of the catalyst; to prove this hypothesis, the same substrate was irradiated in the presence of [Ir(ppy)_3_] (E_T_ = 56.4 kcal/mol). Under these conditions, both double bonds underwent *cis*/*trans* isomerization.

**Scheme 7:**
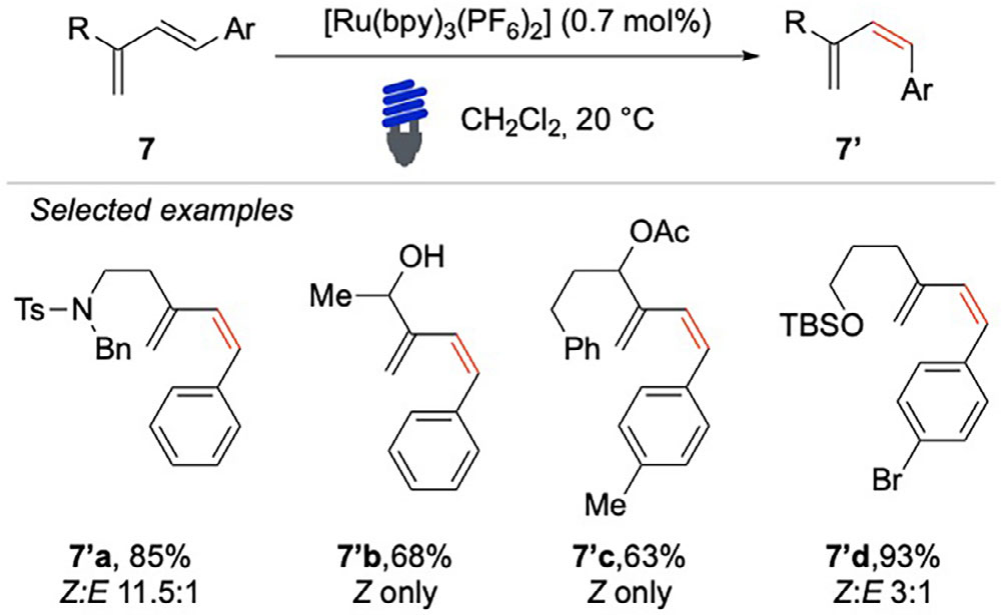
EnT based photo isomerization of functional dienes **7**.

With this concept on their minds, Diver and colleagues addressed the scarcity of methods for the photoisomerization of acyclic 1,3‐dienes.^[^ ^60^ ^]^ This study demonstrated that a judicious choice of the photocatalyst allows the *cis*/*trans* photoisomerization of 1‐aryl‐1,3‐dienes **7** under mild conditions (Scheme 7). By carefully tuning the electronic and steric properties of the catalyst, they achieved high levels of selectivity and efficiency. This provided a valuable tool for accessing *Z*‐configured dienes, which are important intermediates in organic synthesis, particularly in the development of bioactive compounds and materials chemistry, for instance via [4 + 2] cycloaddition. The ruthenium‐based catalyst [Ru(bpy)_3_(PF_6_)_2_] (*E*_T _= 46.5 kcal/mol) demonstrated significantly higher efficiency in the *E*→*Z* photoisomerization process compared to iridium‐based photocatalysts. This can be attributed to its lower triplet energy (45–47 kcal/mol) compared to iridium‐based catalysts (55–60 kcal/mol), which minimized unwanted side reactions and enhanced the overall reaction efficiency.

Remarkably, this method is tolerant to protected amine **7′a**, free hydroxyl groups **7′b**, useful allylic esters **7′c**, and it is compatible with substrates containing aryl bromides **7′d**. Notable feature of this approach is its short reaction time, typically requiring only 20 minutes of irradiation to reach the photo‐stationary state. The enrichment of the *Z*‐isomer is attributed to the *E*_T_ of the photocatalyst. The authors suggest that photocatalysts with higher *E*_T_, such as iridium‐based photosensitizers, lack the selectivity needed to exclusively excite the *E*‐isomer. In contrast, ruthenium‐based photosensitizers, which have a lower *E*_T_, enable the selective activation of the *E*‐isomer, ultimately leading to the accumulation of the *Z*‐isomer. The crucial role of selecting the appropriate photocatalysts with coherent *E*_T_ can limit the generality of the reaction.

**Scheme 8:**
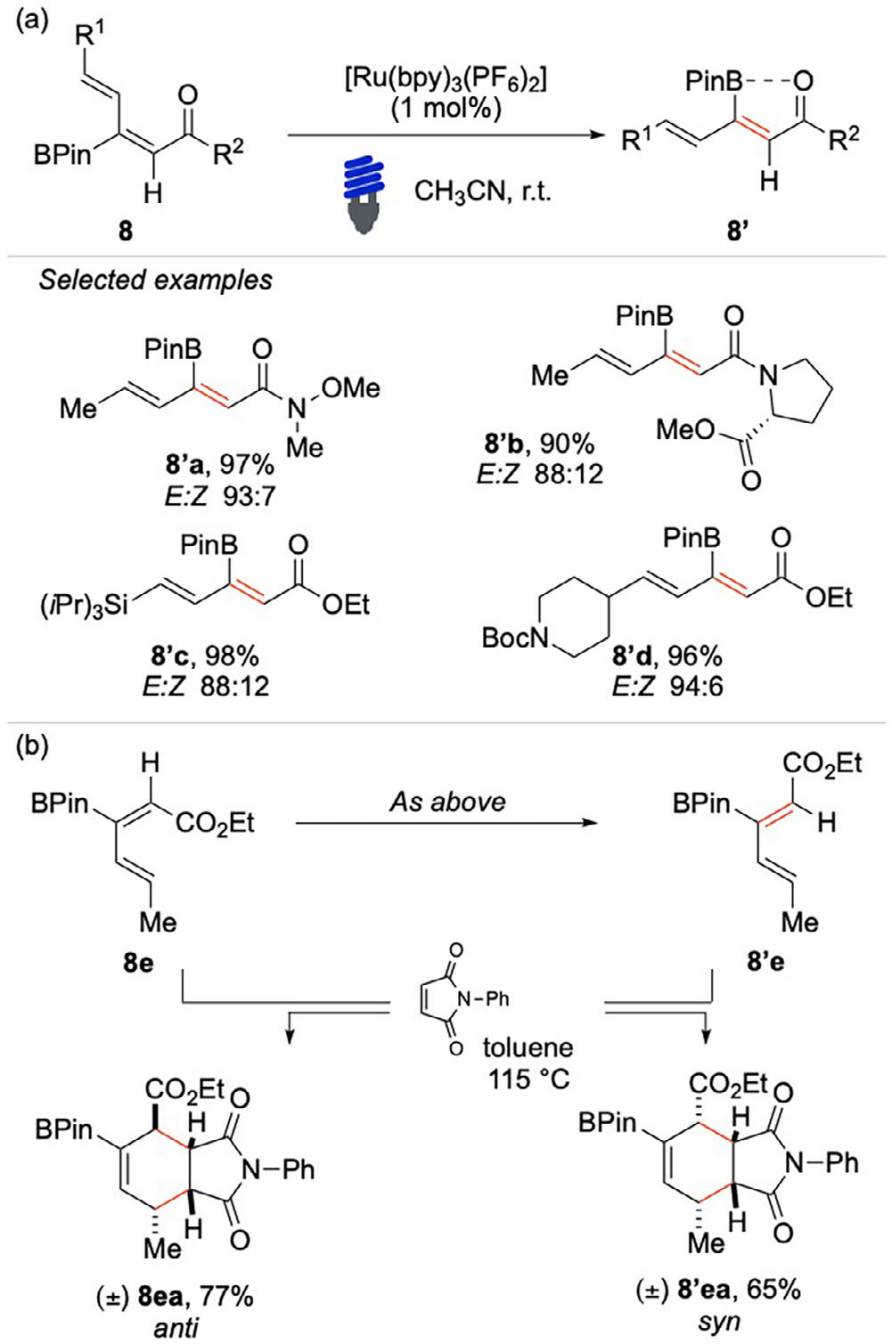
EnT‐based photo isomerization of dienes **8** by Gilmour.

Gilmour and colleagues highlighted how the fine‐tuning of the photocatalyst *E*_T_ ensured the preferential excitation of the *E*‐isomer, promoting the desired photoisomerization.^[^ ^61^ ^]^ By carefully adjusting the photocatalyst properties, they were able to optimize both reaction efficiency and selectivity. In their elegant study, they reported the isomerization of borylated 1,3‐dienes **8** under visible light irradiation (Scheme 8a). Recognizing the synthetic value of these structural motifs, the authors selected sorbic acid‐derived frameworks as a versatile platform for the stereocontrolled modulation of the dienone scaffold, which is a widely utilized motif in natural product synthesis. The (2*Z*)‐1,3‐dienes were conveniently synthesized via hydroboration of the corresponding alkynes, providing an efficient and modular approach to these valuable intermediates. Subsequent photoisomerization was achieved by irradiating the substrates **8** with blue LEDs in the presence of [Ru(bpy)_3_(PF_6_)_2_] (*E*_T_ = 46.6 kcal/mol). The choice of this ruthenium‐based photocatalyst was critical, as its triplet energy selectively promotes the desired *cis*/*trans* isomerization without triggering undesired side reactions. This method demonstrates broad substrate scope, accommodating a variety of substitution patterns while consistently delivering high *E*/*Z* selectivity. Notable examples of substrates utilized in this study include amino acid derivatives **8′a** and **8′b**, TIPS‐protected alkene **8′c**, and structurally diverse natural product derivatives. The stereochemical outcomes of these transformations were thoroughly investigated through X‐ray crystallographic studies, which provided critical insights into the underlying principles governing stereocontrol. Specifically, in the case of the *Z*‐isomer of the 1,3‐diene, the boron p‐orbital participates in the extended conjugation of the system, influencing the electronic distribution and ultimately dictating the stereochemical course of the reaction. The *E*‐isomer exhibits a distinct structural feature wherein the C─B bond is rotated by approximately 90°. This unique spatial arrangement significantly enhances the nO→pB interaction, a stabilizing effect that ultimately contributes to the higher triplet energy of the product.

The mechanistic pathway underlying this transformation has been rigorously investigated through a combination of absorption spectroscopy, luminescence quenching studies, and electrochemical measurements, all of which collectively confirm the occurrence of an EnT process. Beyond its mechanistic significance, this strategy proved valuable in the derivatization of various borylated dienes, demonstrating substantial synthetic value. Its applicability was further exemplified in stereospecific Diels–Alder cycloadditions (Scheme 8b).

**Scheme 9:**
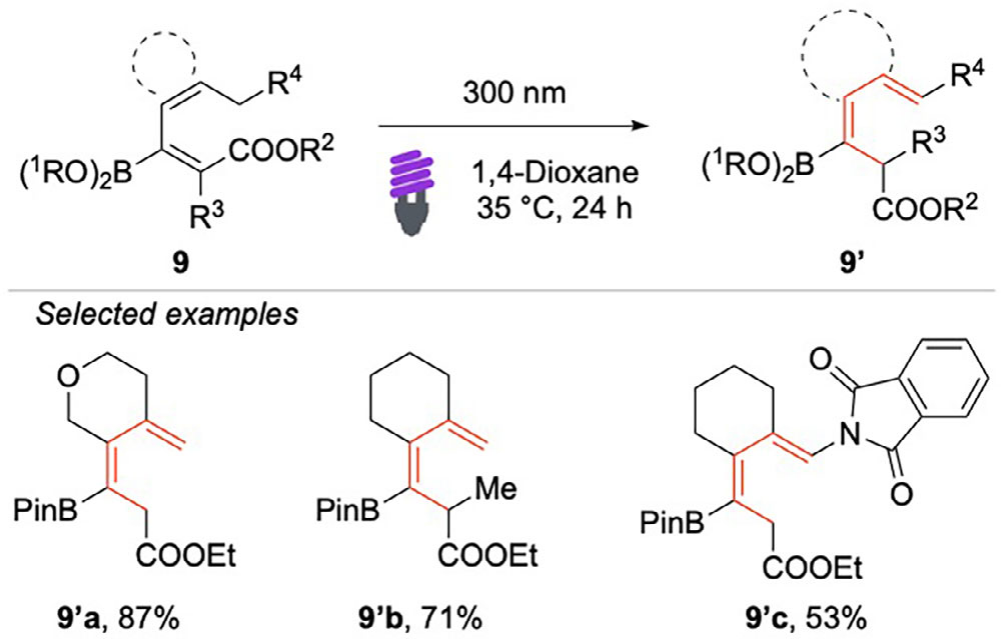
Deconjugative isomerization of borylated dienoates **9**.

Based on a similar approach, Gilmour and coauthors reported a bio‐inspired photochemical strategy for the deconjugative isomerization of borylated dienoates (Scheme 9).^[^ ^62^ ^]^ This strategy proceeds through the direct activation of the borylated dienoates **9** at 300 nm (95.3 kcal/mol). Mechanistic investigations highlighted the importance of both the ester and boron functional groups and suggested a pathway involving an initial contrathermodynamic geometric isomerization, followed by a radical ^[^ ^1^, ^5^ ^]^‐hydrogen migration. The resulting borylated dienes serve as versatile intermediates for further derivatization, underscoring the synthetic utility of this approach.

**Scheme 10:**
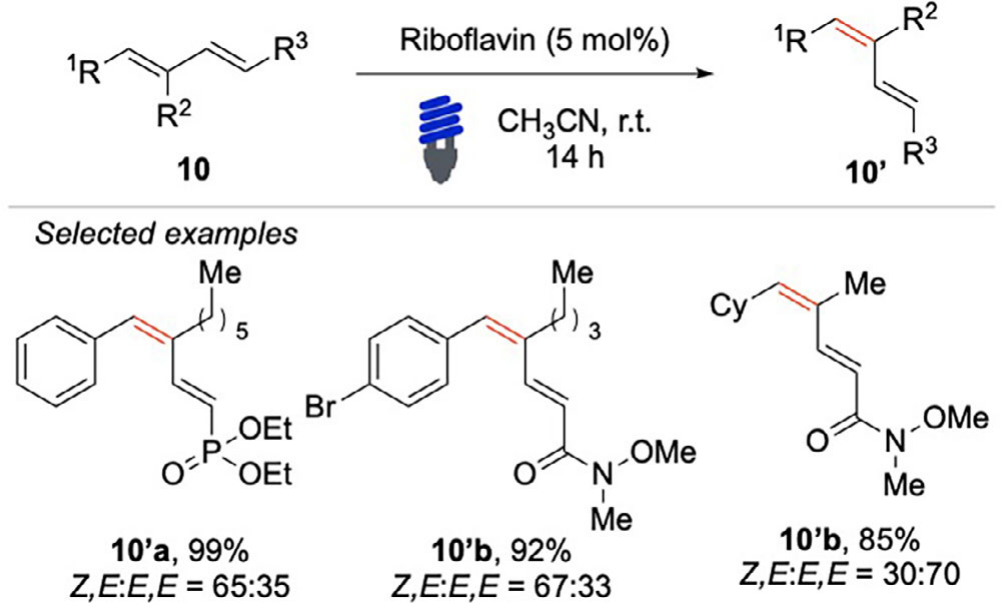
EnTbased photo‐ isomerization of (1E,3E)‐dienes.

Saha, Banerjee, and coworkers have developed a riboflavin‐catalyzed EnT isomerization protocol for (1E,3E)‐dienes **10** (Scheme 10).^[^ ^63^ ^]^ This method demonstrates broad substrate scope, displaying excellent tolerance toward a wide variety of aryl fragments functionalized with diverse electron‐donating and electron‐withdrawing groups. Particularly noteworthy, the protocol allows access to a structurally diverse set of synthetically valuable Weinreb amides.

Since the seminal work by Yoon on the discovery of visible‐light‐mediated photocatalytic EnT [2 + 2] cycloadditions, significant progress has been made in this field as functionalized cyclobutanes play a crucial role in the synthesis of pharmaceuticals and commercially valuable materials.^[^ ^64^, ^65^, ^66^ ^]^

**Scheme 11:**
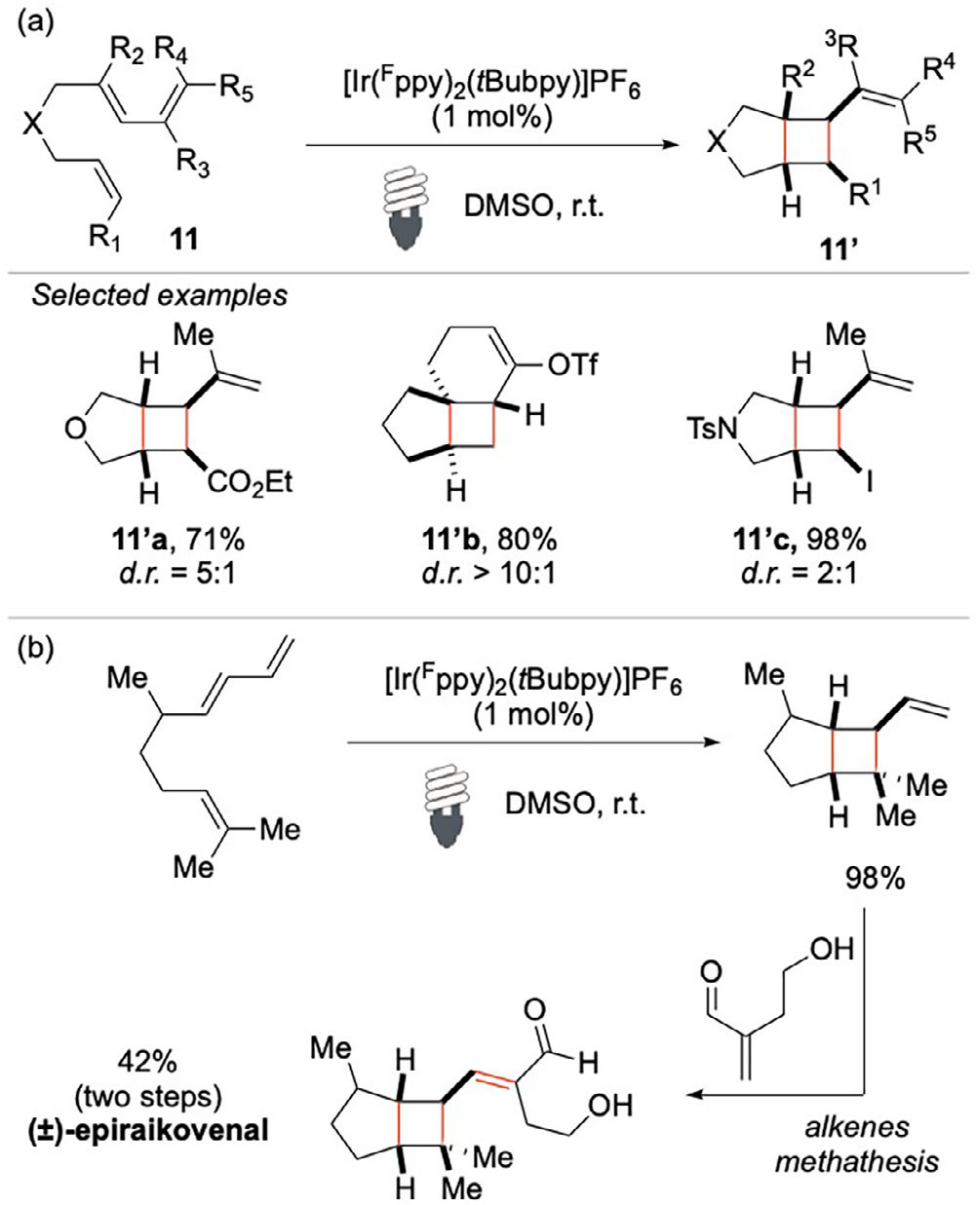
[2 + 2] cycloaddition with dienes **11**.

In his pioneering study, Yoon and his collaborators disclosed a highly efficient [2 + 2] cycloaddition of a diverse range of 1,3‐dienes, a reaction that is facilitated by an iridium‐based photocatalyst under visible‐light irradiation (Scheme 11a).^[^ ^67^ ^]^ The photocatalyst, characterized by an *E*_T_ of 61.8 kcal/mol, effectively populates the triplet excited states of the dienes **11** (*E*_T_ ≃ 60 kcal/mol), thereby enabling their subsequent reactivity and the formation of the desired vinyl cyclobutanes **11′**. A key mechanistic insight proposed by the authors is that this transformation proceeds via an EnT mechanism, rather than a photoredox cycle. To support this hypothesis, they conducted a control experiment using a ruthenium‐based photocatalyst with a significantly lower *E*_T_ (*E*_T_ = 47 kcal/mol), but a similarly convenient oxidation potential (*E*_ox_ = 1.27 V).^[^ ^68^ ^]^ No detectable formation of the [2 + 2] cycloadducts **11′** was observed, strongly reinforcing the notion that the reaction involves a EnT rather than an electron transfer pathway (SET). This method proved to be highly versatile, accommodating a broad range of dienes and functional groups. Oxygen‐tethered dienes participated in the reaction smoothly, affording the corresponding [3.2.0] cycloadduct **11′a** in good yields. Notably, quaternary carbon‐containing products were accessed in the case of **11′b**, demonstrating the utility of this approach for the construction of sterically congested frameworks. One particularly intriguing aspect of this study was the successful incorporation of a vinyl iodide moiety **11′c**, which remained intact throughout the reaction and led to the formation of the desired product in excellent yield.

Beyond its methodological significance, this approach also showcased remarkable potential in natural product synthesis (Scheme 11b). The straightforward total synthesis of the natural product (±)‐epiraikovenal, underscores its practicality for complex molecule construction.

**Scheme 12:**
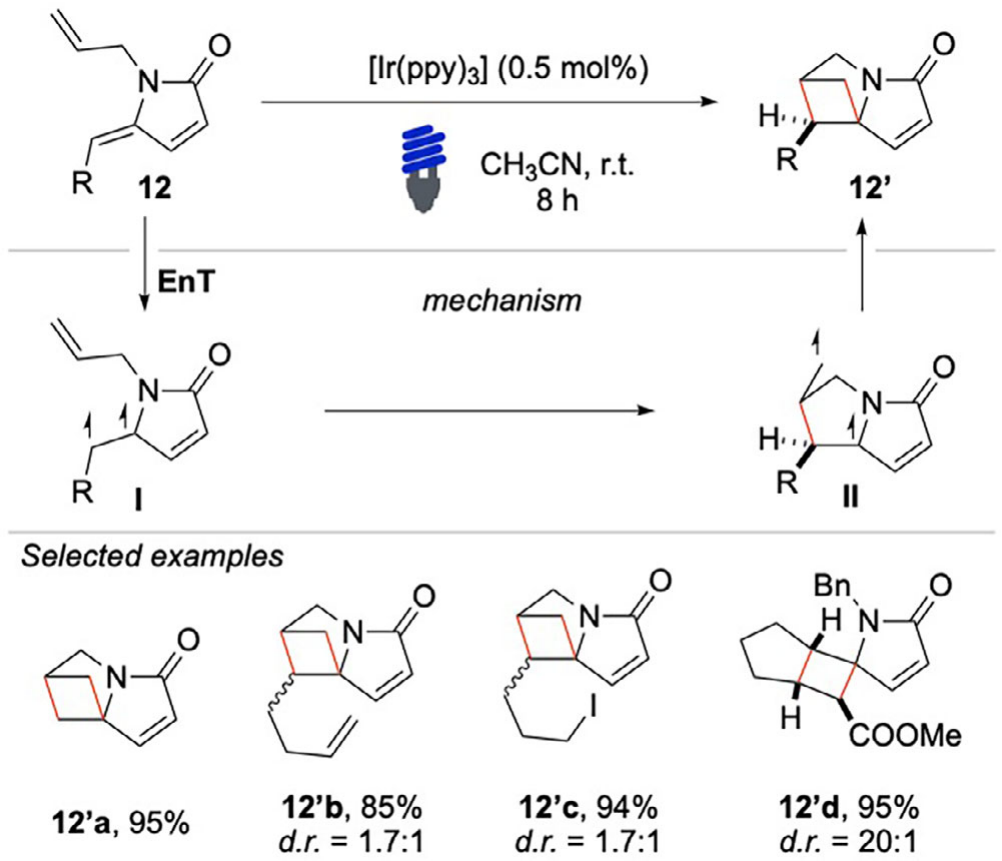
[2 + 2] cycloaddition of unsaturated lactams **12**.

In 2022, Vassilikogiannakis and colleagues reported that the light‐induced EnT activation of γ‐alkylidene‐γ‐lactams **12** could enable the construction of novel sp^3^‐rich spiro‐cycles **12′** (Scheme 12).^[^ ^69^ ^]^ From a mechanistic point of view, upon excitation of compound **12** to its triplet state via EnT, a diradical intermediate **I** is generated. A subsequent *5‐exo‐trig* cyclization occurs, forming the first C─C bond and yielding intermediate **II**, which undergoes sequential ISC and radical recombination, leading to the formation of the second C─C bond and affording the desired product **12′**.

The [2 + 2] photocycloaddition developed in this study involves the use of γ‐lactams, which are highly valuable structural motifs commonly found in natural products and bioactive compounds. Their approach provides a mild, selective, and atom‐economical alternative to traditional synthetic strategies for the synthesis of rigid cyclobutane‐containing scaffolds. The reaction proved to be highly compatible with a broad range of unsaturated lactams, including **12′b** and **12′c**, which bear, respectively, an allylic and iodine moiety, which are useful fragments for additional derivatization. Moreover, the use of **12′d** enabled the formation of a tricyclic framework from a simple precursor, demonstrating the method efficiency in generating complex architectures in a single step.

**Scheme 13:**
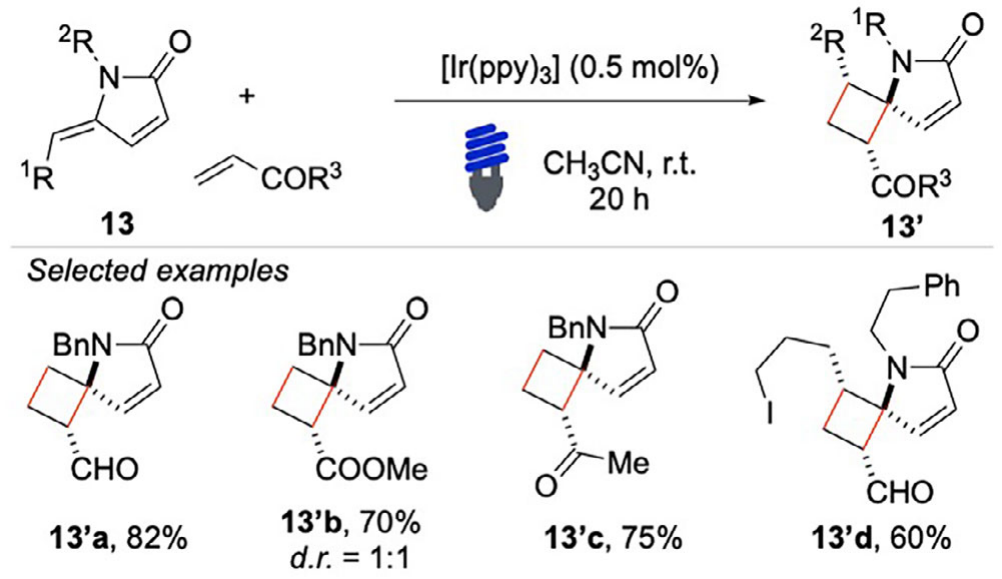
Intermolecular [2 + 2] cycloaddition with diene **13**.

Beyond intramolecular [2 + 2] cycloadditions, the researchers also extended their investigation to more challenging intermolecular reactions (Scheme 13). When γ‐alkylidene‐γ‐lactams **13** reacted with electron‐deficient double bonds, rare 5,4‐spirocycles **13′** were accessible. A variety of α,β‐unsaturated systems, including acrolein **13′a** and acrylic esters **13′b**, were successfully incorporated under the optimized reaction conditions, yielding structurally intriguing and synthetically appealing products.

**Scheme 14:**
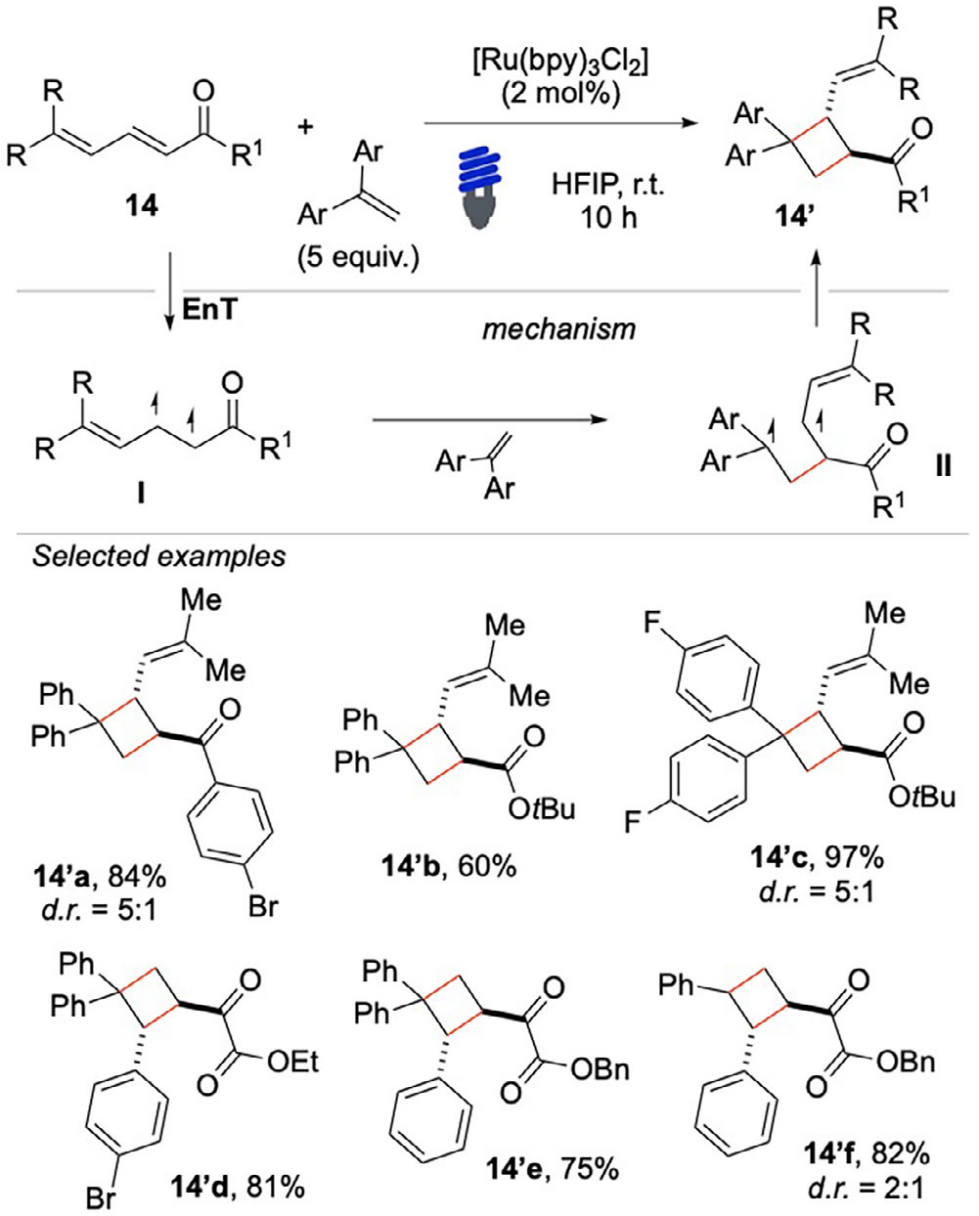
Intermolecular [2 + 2] cycloaddition with 2,4‐dien‐1‐ones **14**.

In 2019, Wu and colleagues reported a selective intermolecular [2 + 2] cycloaddition between 2,4‐dien‐1‐ones **14** and a terminal olefin under visible‐light sensitization (Scheme 14).^[^ ^70^ ^]^

A significant contribution of this study is the mechanistic investigation performed, which included the analysis of electrochemical data and DFT calculations to support that the activation of the substrate occurs via EnT. The computational analysis focused on 2,4‐dien‐1‐ones **14**, revealing that the [2 + 2] cycloaddition predominantly occurs at the C═C bond adjacent to the carbonyl group. This selectivity arises from differences in transition‐state energy barriers, with the C2 position exhibiting the lowest barrier for addition (7.5 kcal/mol), making it the most favorable reaction site. In contrast, the C5 position had a slightly higher barrier (10.7 kcal/mol), rendering it less reactive. Other possible reaction sites exhibited significantly higher barriers, further reinforcing the energetic convenience for the C2 attack over alternative pathways, such as the formation of an oxetane ring.

The use of π‐extended conjugated dienones, which, upon excitation, selectively react at their C2‐C3 positions, represents an innovative and efficient alternative to conventional synthetic routes. Although the reagents **14** needed the presence of two R substituents, alkene and styrene derivatives only were suitable olefinic partners for this reaction, various functionalities were tolerated.

**Scheme 15:**
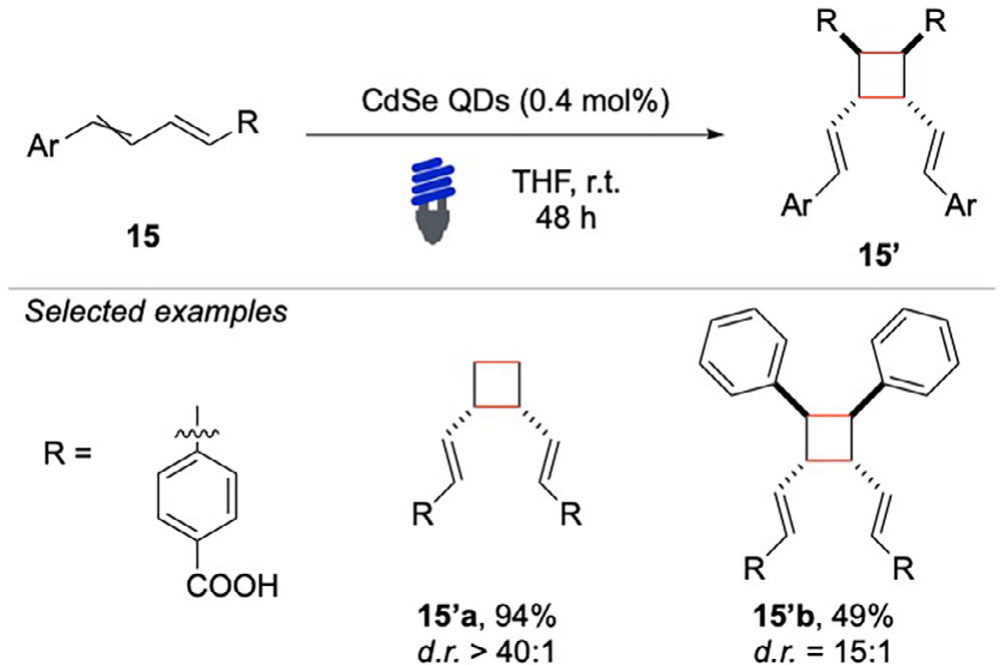
Intermolecular [2 + 2] photocycloadditions of dienes **15**.

Among the various [2 + 2] cycloaddition reactions that have been explored in recent years, particular attention has been directed toward those involving aryl‐alkenes and 1,3‐dienes. In 2021, Weiss and coworkers reported a particularly noteworthy and challenging case of selective [2 + 2] photocycloaddition between two 1,3‐dienes (Scheme 15).^[^ ^71^ ^]^ The challenges associated with this transformation lie in controlling both the chemo‐ and the stereo‐selectivity of the reaction, owing to the presence of multiple conjugated double bonds, which can lead to a variety of competing pathways and undesired side products.

**Scheme 16:**
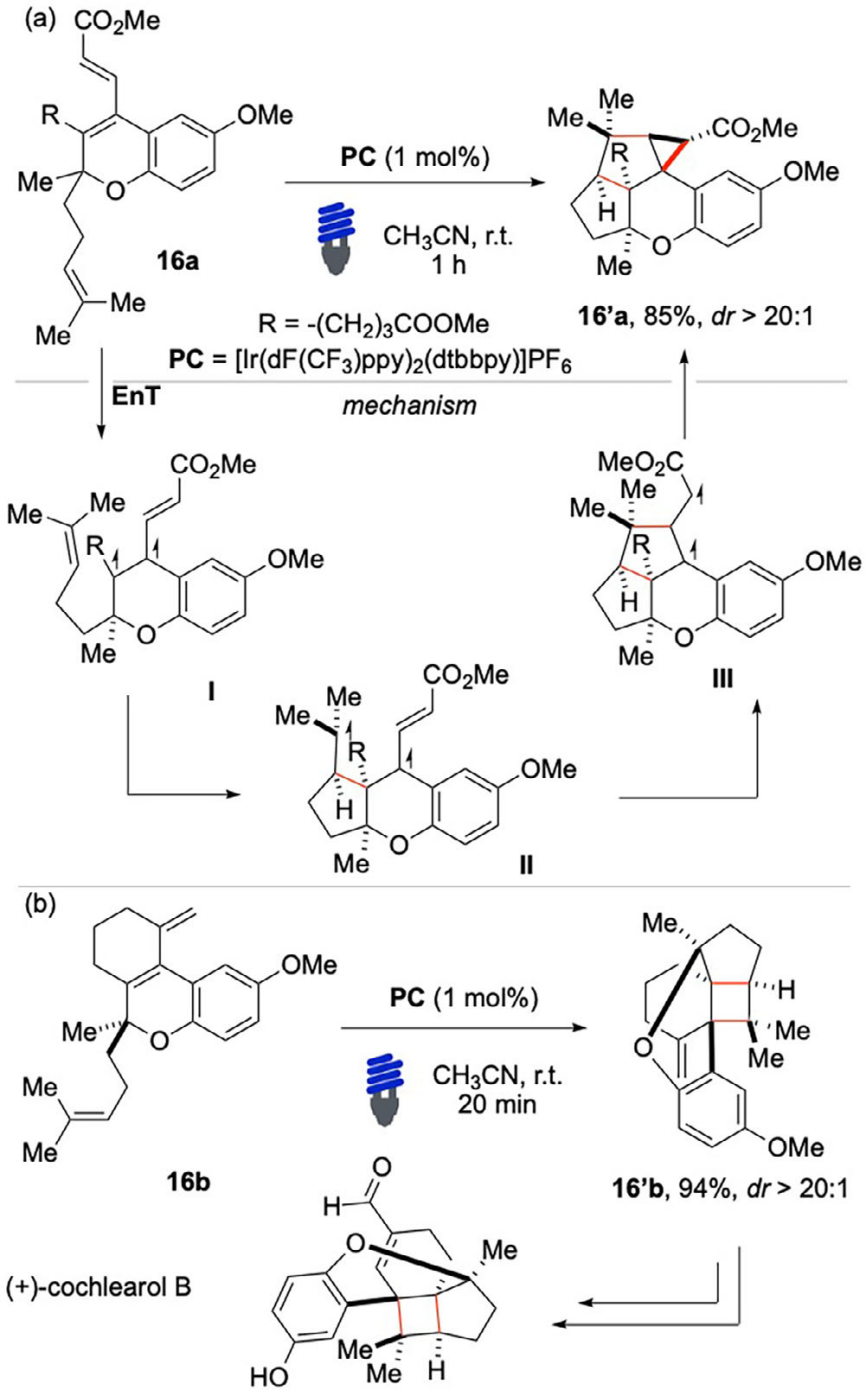
EnT light‐mediated synthesis of (+)‐Cochlearol B.

Initially, several conventional photosensitizers were tested with limited success. Good results were achieved using quasi‐spherical cadmium selenide (CdSe) quantum dots (QDs). These species were found capable of enabling the EnT pathway, whereas more popular photosensitizers unleashed undesired competitive pathways based on electron‐transfer steps, such as [4 + 2] cycloadditions that likely occurred via formation of transient radical cations. Taking into account the broad functional group tolerance and the structural complexity that can be achieved through light‐mediated cycloadditions, Schindler and colleagues, in 2022, devised an innovative total synthesis of (+)‐cochlearol B (Scheme 16).^[^ ^72^ ^]^ Their synthetic strategy was centered around a late‐stage [2 + 2] cycloaddition of highly conjugated diene **16a**, enabled by EnT catalysis. By employing a late‐stage cycloaddition, the researchers were able to construct the complex polycyclic architectures with high efficiency and stereocontrol. The intermediate **16a** was initially synthesized as a key precursor for the monoterpenoid target. However, the reaction unexpectedly led to the formation of the cyclopropane‐containing side product **16′a**. This unforeseen outcome suggested that the electronic and steric properties of the diene played a crucial role in determining the reaction pathway. To overcome this challenge, the researchers strategically modified the electronic characteristics of the diene by synthesizing **16b**, successfully redirecting the reaction toward the desired [2 + 2] cycloaddition **16′b**. This crucial adjustment ultimately enabled the completion of the synthesis of the key intermediate necessary for the final construction of the target compound. These findings highlight the remarkable structural diversity that can be accessed through EnT‐based photochemical processes.

In advance of the construction of highly functionalized compounds through simple and practical approaches, dearomatization reactions have gained significant attention due to their crucial role in accessing complex 3D structures.^[^ ^18^, ^73^ ^]^ These transformations enable the efficient conversion of readily available aromatic compounds into architecturally intricate frameworks, expanding the scope of molecular diversity in synthetic chemistry.^[^ ^74^ ^]^

**Scheme 17:**
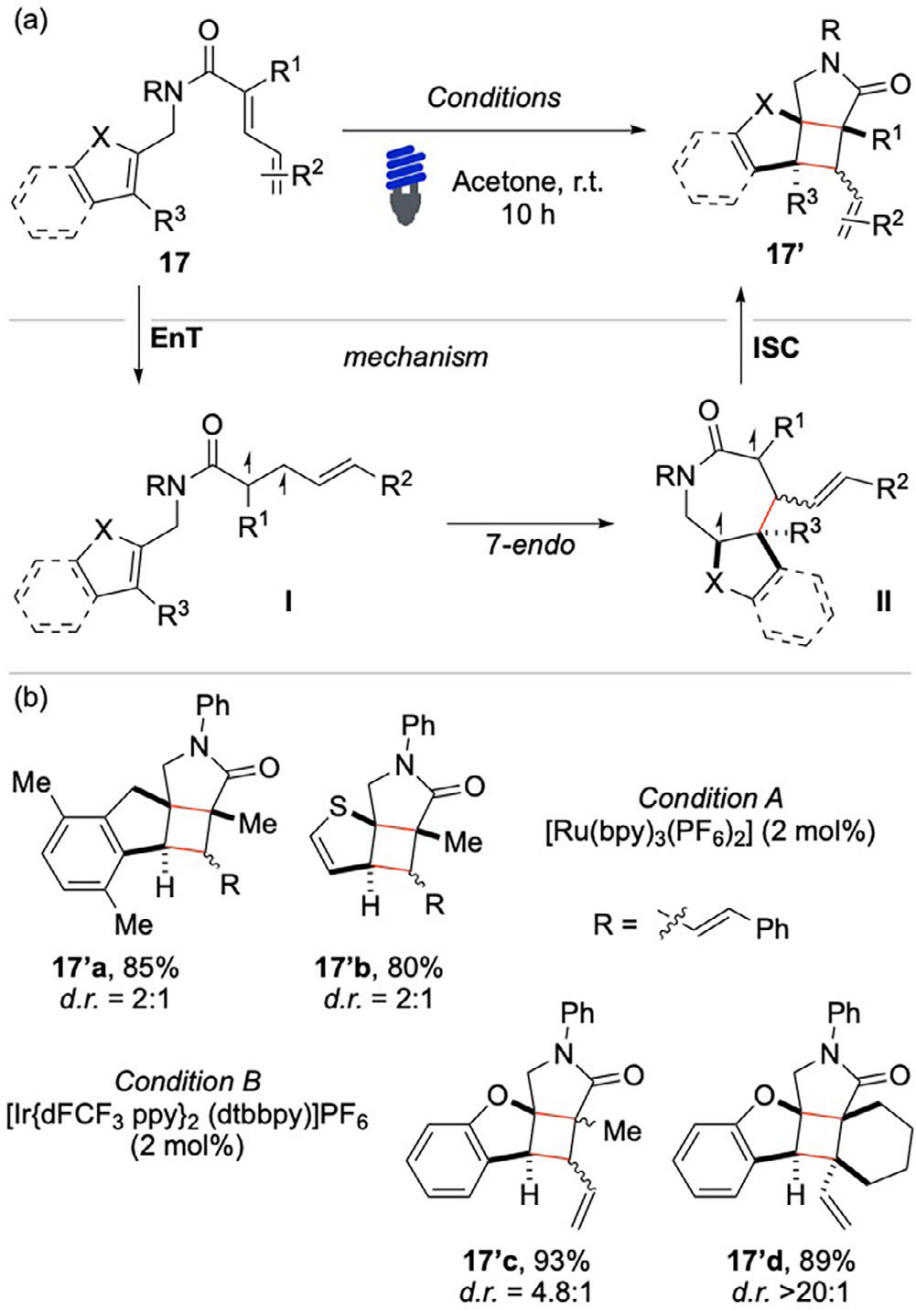
Dearomative [2 + 2] with styryl dienes and dienes.

In this context, Xu and colleagues made a significant contribution in 2023 by reporting a novel diastereoselective cyclization strategy aimed at constructing multiple contiguous quaternary carbon stereocenters (Scheme 17).^[^ ^75^ ^]^ This transformation begins with the sensitization of the dienamide **17** to its triplet excited state **I**, followed by a *7‐endo‐trig* cyclization that leads to the formation of intermediate **II**. Although a *5‐exo* cyclization could also be envisioned for the presented cyclization, the authors proposed that the radical generated through the formation of the first C─C bond is stabilized by the presence of the oxygen atom in the α‐position, favoring the *7‐endo* cyclization. The intermediate **II**, upon ISC, can subsequently recombine to afford the final product **17′**. The major stereochemical outcome of the reaction is primarily attributed to steric hindrance, which dictates the predominant configuration of the product. Notably, because intermediates **I** and **II** have the potential to epimerize, this dynamic equilibrium makes the observed diastereoselectivity even more remarkable. The method resulted applicable to various heterocycles, containing simple five‐membered oxygen‐ and sulfur‐containing aromatic systems, such as furan and thiophene **17′b** and more complex nitrogen‐based scaffolds, including *N*‐methylindole and benzofurans **17′d**. The reaction proceeds with remarkably high yields and, in many cases, exhibits excellent levels of diastereoselectivity. Although demonstrated with only two examples, the reaction was further extended to include additional dienes **17′b** and **17′d**, which have reduced conjugation by removing an aryl ring, by employing a more potent EnT sensitizer.

Significant advances have been achieved in EnT for 1,3‐diene isomerization and cycloaddition, particularly in total synthesis. However, challenges remain, including the achievement of energy efficiency at scale and the investigation of continuous‐flow setups. At the same time, there are abundant opportunities to enhance molecular complexity and selectivity, further realizing the potential of light‐mediated EnT catalysis. Continued exploration of these avenues promises to expand the synthetic capabilities of visible‐light‐driven transformations in both academic and industrial settings.

## 3 Activation of Cumulated 1,2‐Dienes

Allenes have been extensively employed in organic chemistry owing to their distinctive structural and electronic features, which confer a unique reactivity profile that clearly sets them apart from conventional alkenes and alkynes.^[^ ^76^, ^77^ ^]^ Unlike simple unsaturated hydrocarbons, allenes contain a cumulated π‐system composed of two orthogonal double bonds centered on a single sp‐hybridized carbon atom. This unusual bonding arrangement imparts exceptional physical and chemical properties that continue to intrigue chemists and stimulate the development of novel synthetic methodologies.

The central carbon atom, which connects two mutually perpendicular π‐bonds, not only imposes strict linearity on the allene framework but also introduces inherent axial chirality when the substituents on each terminus differ.^[^ ^78^, ^79^, ^80^ ^]^ The stereoelectronic effects arising from the cumulated π‐system play a central role in dictating the reactivity of allenes. Their unique orbital interactions enable reaction pathways that are often inaccessible for traditional alkenes or alkynes.^[^ ^81^, ^82^, ^83^ ^]^ These stereoelectronic influences can be harnessed to design new transformations that deliver specific regio‐ and stereo‐chemical architectures.

The versatile reactivity of allenes has been extensively explored across a diverse range of synthetic transformations. They readily participate in cycloaddition reactions, providing efficient access to carbo‐ and heterocyclic frameworks with high levels of structural diversity. Furthermore, their π‐systems are susceptible to both nucleophilic and electrophilic additions, often proceeding with exceptional chemo‐ and regio‐selectivity.^[^ ^84^ ^]^ Transition‐metal‐catalyzed processes involving allenes have also emerged as a particularly rich area of research, enabling C─C and C─heteroatom bond formation through mechanisms that exploit the unique electronic characteristics of the cumulated double bonds. In addition, radical‐mediated functionalizations of allenes have broadened the synthetic toolbox, offering new strategies for late‐stage diversification and the assembly of complex molecules.^[^ ^85^, ^86^ ^]^

Over the last few decades, radical chemistry has undergone a tremendous evolution, enabling the development of visible‐light‐promoted processes.^[^ ^9^ ^]^ Today, countless functional groups can be activated to generate radical intermediates.^[^ ^87^ ^]^ In particular, the generation of biradical intermediates under mild conditions has opened up broad synthetic opportunities.

Although initial studies have demonstrated the use of allenes as coupling partners in visible light‐mediated reactions, the present review will specifically focus on the strategies and mechanisms that involve the activation of allenyl fragments, highlighting their potential in photochemical transformations. ^[^ ^87^, ^88^, ^89^, ^90^, ^91^ ^]^

**Scheme 18:**
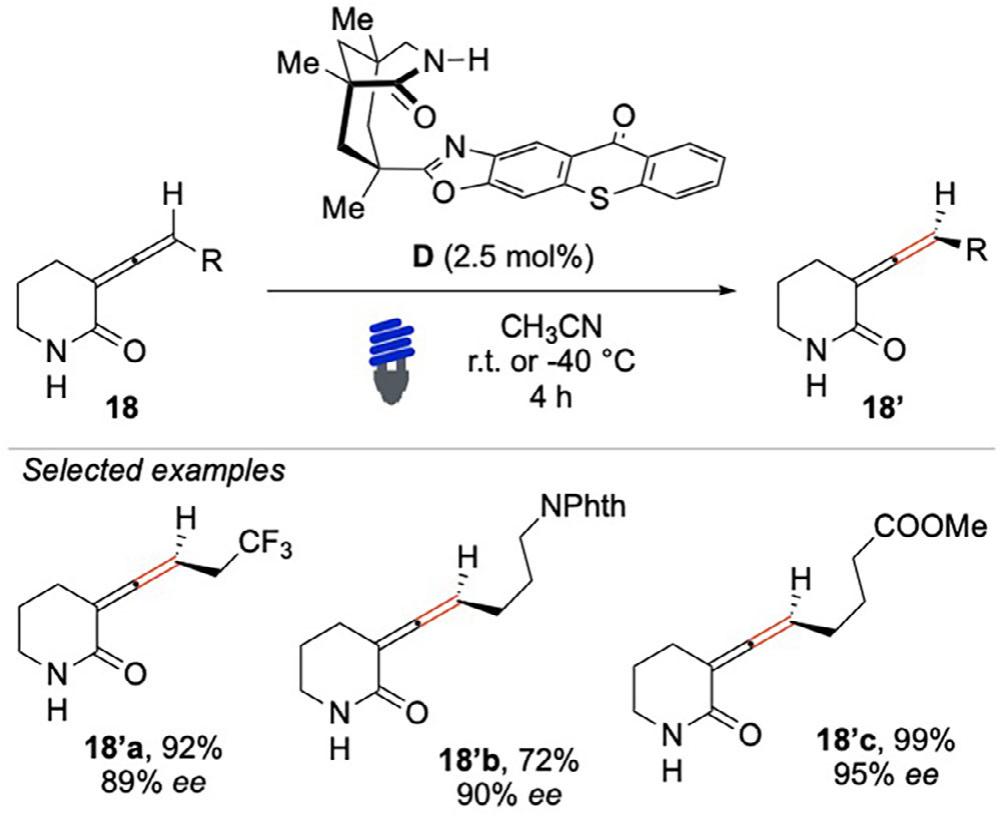
Light‐mediated deracemization of *C*‐allenamides **18**.

In 2018, Bach and colleagues tackled the challenging deracemization of poorly activated allenes **18** by integrating hydrogen bond recognition with EnT photocatalysis (Scheme 18).^[^ ^92^ ^]^ This innovative approach was possible thanks to the use of a conformationally rigid photocatalyst, the lactam **D**, which served as a chiral hydrogen bond donor, tethered to a modified thioxanthone sensitizer.

The thioxanthone moiety played a crucial role in sensitizing the tri‐substituted allene. Moreover, the strong bi‐directional hydrogen‐bonding interaction between the amide group of the catalyst and that of the six‐membered lactam substrate ensured a highly controlled ISC. This stereoselective transition ultimately resulted in the preferential enrichment of one axial enantiomer. The methodology proceeded under mild conditions, requiring only a low catalyst loading and operating effectively at low temperatures. The demonstrated remarkable efficiency furnished chiral 2‐piperidone allenes **11** with high enantioselectivity, good yields, and excellent functional group tolerance. Indeed, the method can be applied to a diverse range of substrates, such as amines **11b’** and esters **11c’**. A crucial aspect influencing the reaction outcome was the size of the lactam, which significantly affected the formation of the key hydrogen bond network.

**Scheme 19:**
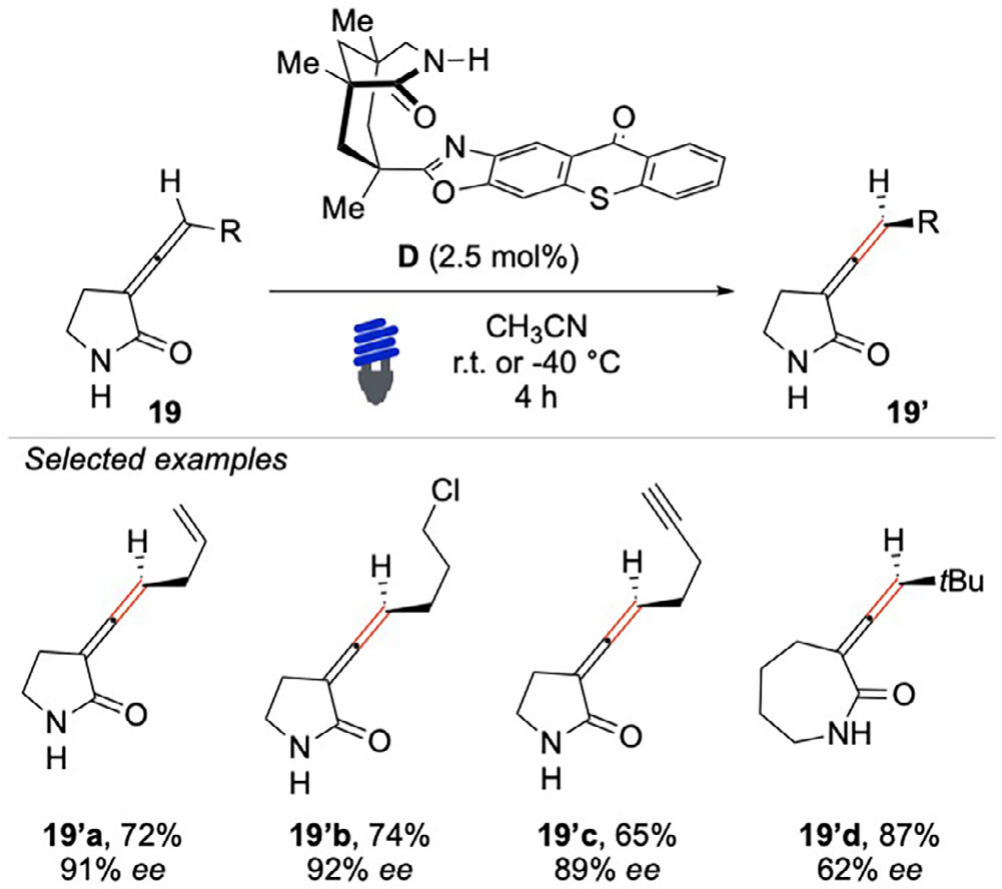
Light‐mediated deracemization of *C*‐allenamides **19**.

Given the impact of the lactam ring size, the same research group conducted a follow‐up study to investigate this structural factor in greater depth (Scheme 19).^[^ ^93^ ^]^ Their findings revealed the critical role of ring size in facilitating effective hydrogen bonding and, thus, the desired catalytic efficiency. Functionalized pyrrolidones **19** emerged as promising substrates, demonstrating their suitability for the deracemization reaction. Interestingly, these substrates not only get a comparable functional group tolerance but also allow even higher overall yields and enantioselectivities compared to their six‐membered analogs. Alternatively, an enriched seven‐member ring was also obtained, albeit with a moderate *ee*. Extending the investigation to the deracemization of tetrasubstituted allenes, the optical performance was noticeably inferior, suggesting that additional steric and electronic factors may influence the effectiveness of the photocatalytic EnT approach applied to more complex allene derivatives.

**Scheme 20:**
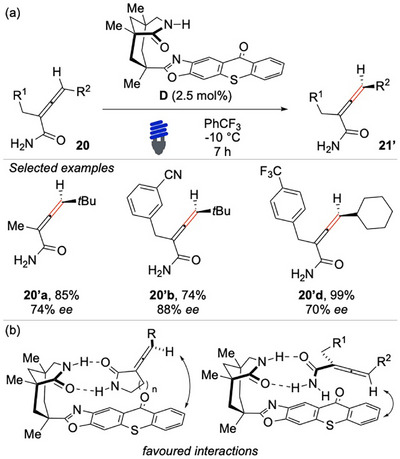
Light‐mediated deracemization of acyclic primary amides **20**.

In 2021, the research group further expanded the scope of this concept by successfully applying it to acyclic primary amides (Scheme 20a).^[^ ^94^ ^]^ These substrates pose a greater challenge compared to their cyclic counterparts, primarily because of the lower control of the stereospecificity in the hydrogen bond network that can be formed with the catalyst **D**. Additionally, the intrinsic flexibility of the acyclic system is an issue of concern for a deracemization method. Despite these threats, acyclic primary amides represent a major leap forward for the subsequent transformations of enantioenriched allenes into a wider array of valuable chiral molecules.

Employing the catalyst **D**, the researchers were able to isolate a diverse range of functionalized enantioenriched allenes **20′**. As a direct consequence of the conformational flexibility, the enantio‐determining step was found to be less efficient. Nonetheless, good enantiomeric excesses were still achieved when bulky substituents were present at both the 2‐ and 4‐positions of the allene, as for **20′a** and **20′b**. A variety of challenging functional groups were well tolerated under the reaction conditions, leading to isolation of the enantioenriched allene products in good to high yields, thus underscoring the robustness and generality of the methodology. The fundamental aspect that governs the success of the deracemization process is the exceptionally high affinity between the catalyst and the amide functional group of the substrate (Scheme 20b). The dual‐catalytic system operates by engaging in hydrogen bonding with the amide moiety, effectively stabilizing the substrate in a well‐defined conformation. This precise spatial arrangement is crucial to direct the stereochemical course of the reaction, particularly during the ISC, in which the excited‐state species undergoes relaxation. The bi‐radical intermediate formed during this process is guided through a stereoselective relaxation pathway that ultimately favors the formation of one enantiomer over the other.

**Scheme 21:**
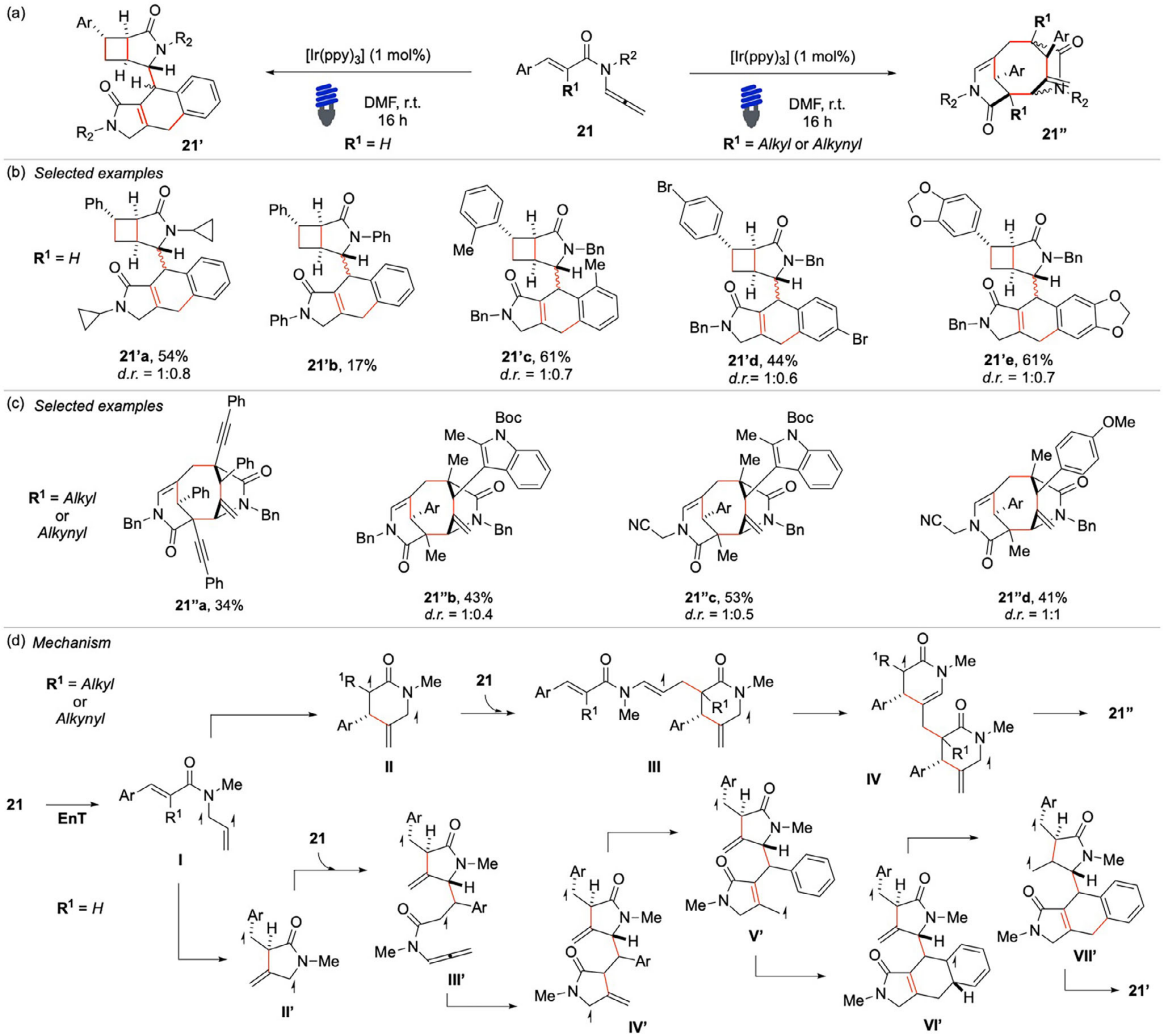
Divergent visible‐light‐promoted dimerization of en‐*N*‐allenamides **21**.

Building on our interest in radical cascade reactions triggered by visible light, in 2022, our group developed the first visible‐light‐promoted dimerization of enallenamides.^[^ ^95^, ^96^, ^97^ ^]^ In this study, a library of en‐*N*‐allenamides **21** in the presence of the commonly employed photocatalyst [Ir(ppy)_3_] led to two distinct reactions (Scheme 21). For substrates in which the R^1^ group is a hydrogen atom, the reaction yielded a [3.2.0] bicyclic unit tethered to an intricate fused tricyclic framework (**21′**), with yields ranging from 17% to 63% (Scheme 21a). Nitrogen substitutions were well tolerated, and alkyl substituents proved significantly more effective than aryl groups. The latter led to a considerable reduction in yield, with compound **21′b** obtained in only 17% yield. The arene component of the styryl fragment exhibited a broad functional group tolerance, accommodating both electron‐donating and electron‐withdrawing substituents at various positions. These results underscore the robustness and versatility of this visible‐light‐induced dimerization strategy for the construction of molecular complexity.

Conversely, when R^1^ is an alkyl or alkynyl moiety, an alternative radical cascade pathway is triggered, leading to the formation of a structurally distinct bridged tricyclic scaffold (**21′’**) in yields ranging from 31% to 64% (Scheme 21b). Notably, the core of the bridged polycycle features a central cyclooctane ring with two headbridging quaternary carbons and an exocyclic double bond, reminiscent of the characteristic B‐ring of the natural taxanes.^[^ ^97^ ^]^ The reaction exhibits remarkable functional group tolerance, accommodating a variety of electron‐donating and electron‐withdrawing substituents. An intriguing aspect of these transformations is their exceptional stereoselectivity. The two distinct product classes are consistently obtained as a mixture of only two diastereomers, a noteworthy outcome given the complexity of the process. Moreover, up to five new carbon‐carbon (C─C) bonds are forged in a single step, achieving complete atom economy.

Mechanistic investigations included deuterium‐labeling experiments and DFT calculations to elucidate the complementary reaction mechanisms (Scheme 21d). Upon EnT activation of the allenamide **21**, the intermediate **I**, which has a diradical character, is formed. Notably, the *E*_T_ of **I** is more than 4 kcal/mol lower than that of a species in which the two mono‐occupied molecular orbitals are localized on the carbon nuclei of the cinnamoyl moiety. This energetic difference was surprising because the sensitization of N‐allenamides was not previously reported, while the EnT activation of styryl‐ and cinnamoyl‐ arms is well established.

Following sensitization, intermediate **I** can undergo cyclization through two distinct pathways, depending on the nature of the substituent at the R^1^ position, thereby initiating one of the two divergent sequences. When the α‐position is unsubstituted, the reaction proceeds via an initial 5‐exo‐trig cyclization, generating intermediate **II′**. This species then engages in a polarity reversed radical addition onto another molecule of **21**, leading to the formation of the α‐acyl radical intermediate **III′**. Subsequently, a 5‐exo‐dig cyclization occurs, producing intermediate **IV′**, which undergoes further transformations via two successive HAT events to generate **V′**. A subsequent *6‐endo‐trig* cyclization furnishes **VI′**, which, following a favorable 1,5‐HAT, gives rise to **VII′**. Finally, through an ISC, the [3.2.0] bicyclic unit teth ered to a fused tricycle of **21′** is generated. Alternatively, when the R1 position is substituted, the reaction follows a distinct trajectory. In this case, intermediate I preferentially adds onto the least hindered terminus of **21**. The resulting intermediate undergoes a sterically controlled (‐endo‐trig cyclization, leading to the formation of **IX**. This species then undergoes further structural rearrangement, culminating in the formation of inter mediate **X**, which facilitates the closure of the central cyclooctane ring through ISC, ultimately affording the bridged tricyclic product **21**″.

**Scheme 22:**
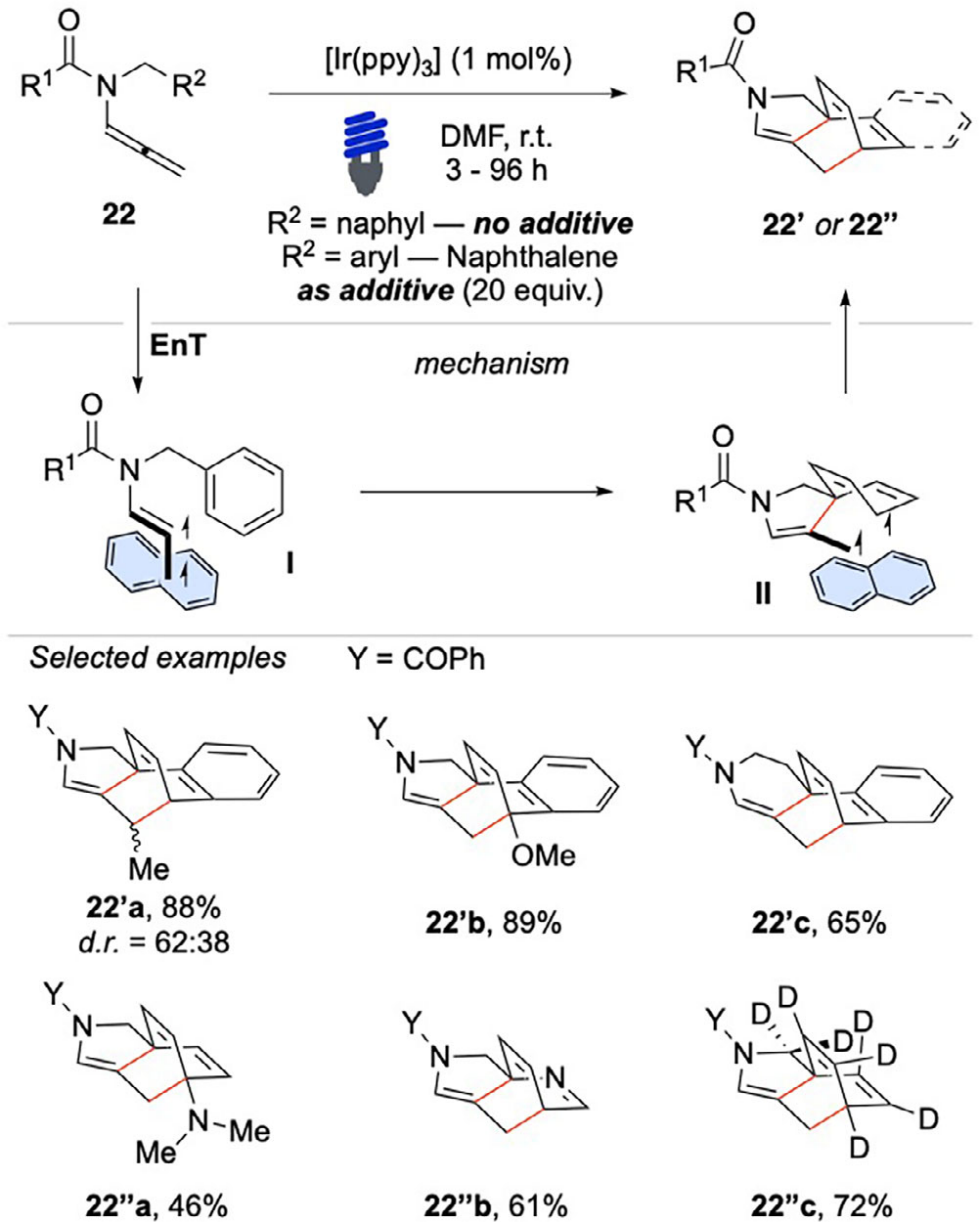
Intramolecular dearomatization of benzyl and naphthyl arms upon activation of N‐allenamides **22**.

In 2023, we introduced an intramolecular dearomatization strategy that targets naphthyl and benzyl groups via light‐mediated activation of allenamides, thereby expanding the synthetic toolbox for constructing complex 3D sp^3^‐rich architectures.^[^ ^99^ ^]^ Exposing the allenamides **22** to visible light in the presence of [Ir(ppy)_3_], the dearomatization of naphthyl or benzyl substrates proceeded efficiently, delivering the product **22′** in consistently high yields (Scheme 22a).

The cascade reaction begins with the successful sensitization of **22** to its triplet state **I**. The highly reactive vinyl radical site of **I** drives the cyclization event, adding to the ipso carbon of the arene, disrupting its aromaticity and forming the first C─C bond. The readily formed intermediate **II** is stabilized especially in the case of naphthyl‐containing substrates. Upon ISC, the diradical recombination forms the second C─C bond affording the desired product **22′**. The robustness of the procedures was proved and terminal substituted allene **22** exhibited a particularly high yield and favorable diastereoselectivity, while substitutions on the naphthalene core were well tolerated without significant loss of efficiency (Scheme 22b).

The method enabled the formation of structurally valuable frameworks, such as the closure of a tetrahydropyridine ring system **22′c**, which could be particularly useful in medicinal chemistry and drug discovery. Significantly more challenging, because of the intrinsic endergonic nature of the reaction, proved to be the dearomatization of benzyl moieties. The feat was made feasible thanks to the addition of naphthalene.

A combination of DFT calculations and experimental validation provided strong evidence about the fundamental role of naphthalene as a co‐catalyst, which can stabilize the triplet intermediates of the sequence via radical‐dispersion interactions, greatly facilitating the desired transformation.^[^ ^97^ ^]^ The additive plays a crucial role in the benzyl pathway by stabilizing all intermediates and the final product by up to 4 kcal/mol, thus preventing the reverse reaction of **II** that would provide back the starting material.

The functional group tolerance highlighted that substitutions on the benzyl scaffold were well tolerated and even substrates bearing electronically distinct and sterically demanding functional groups performed effectively. Highly oxidizable tertiary amines **22′’a** and pyridine functionalities **22′’b** were compatible with the reaction conditions. Notably, a deuterium‐labeled polycyclic product **22′’c** was obtained in an appreciable yield of 72%, showcasing the potential utility of this approach for the synthesis of isotopically labeled compound.

**Scheme 23:**
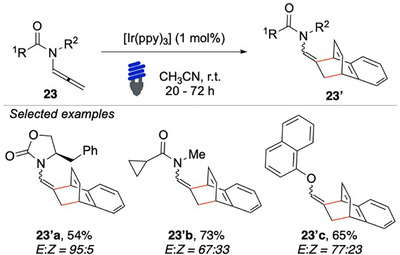
Intermolecular dearomatization of naphthalene with *N*‐allenamides **23**.

Later on, our group disclosed the more challenging intermolecular dearomative para‐cycloaddition on naphthalene (Scheme 23).^[^ ^100^ ^]^ By using [Ir(ppy)_3_] as a photosensitizer under visible light, this study focused on the reactions of allenamides **23** with naphthalenes. The reaction proved to be robust and scalable up to 1 mmol, maintaining a high yield. Substituted naphthalenes could be coupled, although the regioselectivity could be improved, as seen in the formation of **23′a**.

Additionally, the reaction with allenol was successfully tested, providing **23′c** in a promising yield of 65%. These results open up possibilities for testing allenols in further chemical transformations, as they can be activated similarly to *N*‐allenamides.

**Scheme 24:**
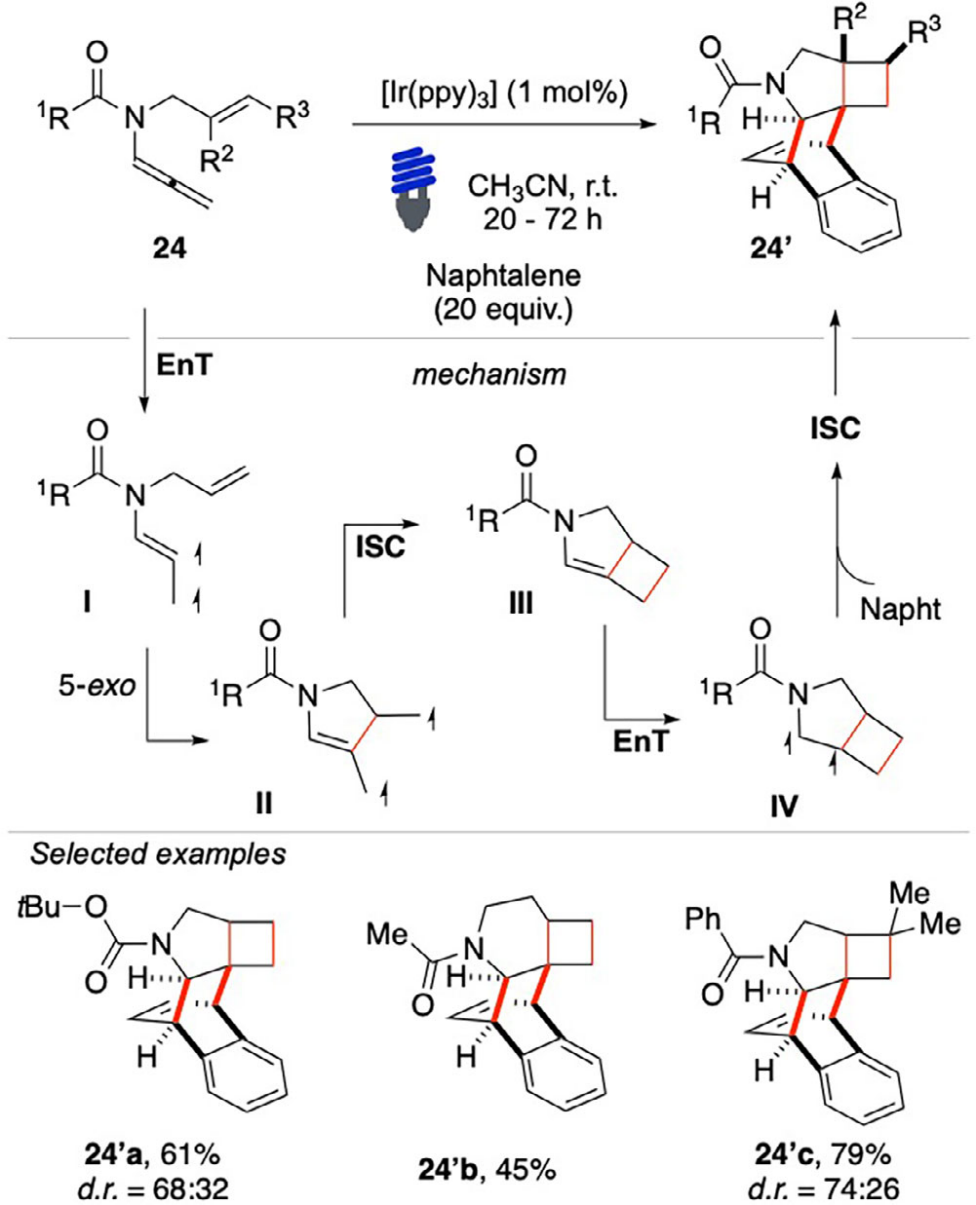
Intermolecular dearomatization of naphthalene with enallenes **24**.

When the amide was substituted with an additional allyl moiety, a novel cascade reaction was achieved (Scheme 24a). This reaction led to the formation of the congested, fused/bridged pentacyclic product **24′**, which was obtained as a mixture of just two diastereomers. This cascade reactivity is particularly significant as it results in the formation of four new carbon‐carbon bonds and up to six contiguous stereocenters.

Stern–Volmer quenching studies, along with DFT calculations, validated the proposed reaction mechanism.^[^ ^96^ ^]^ The formation of the **24′** products follow a distinct cascade pathway. The process begins with the sensitization of substrate **24** to its triplet excited state via EnT from the excited photocatalyst. The intermediate **I** undergoes an intramolecular [2 + 2] cycloaddition with the alkene moiety, leading to the formation of the bicyclo [3.2.0] intermediate **III**. Subsequently, upon a second sensitization, triplet **IV** attacks naphthalene. The process proceeds similarly to the intramolecular dearomative para‐cycloaddition pathway presented above, ultimately yielding the desired class of products **24′**. The reaction exhibited excellent functional group tolerance, enabling the use of Boc‐protected amide **24′a**, the formation of a sealed piperidine central ring **24′b** and of various alkene moieties, including a crotyl arm as in **24′c**, with good to excellent yields, especially considering the complexity of the cascade process (Scheme 24b).

**Scheme 25:**
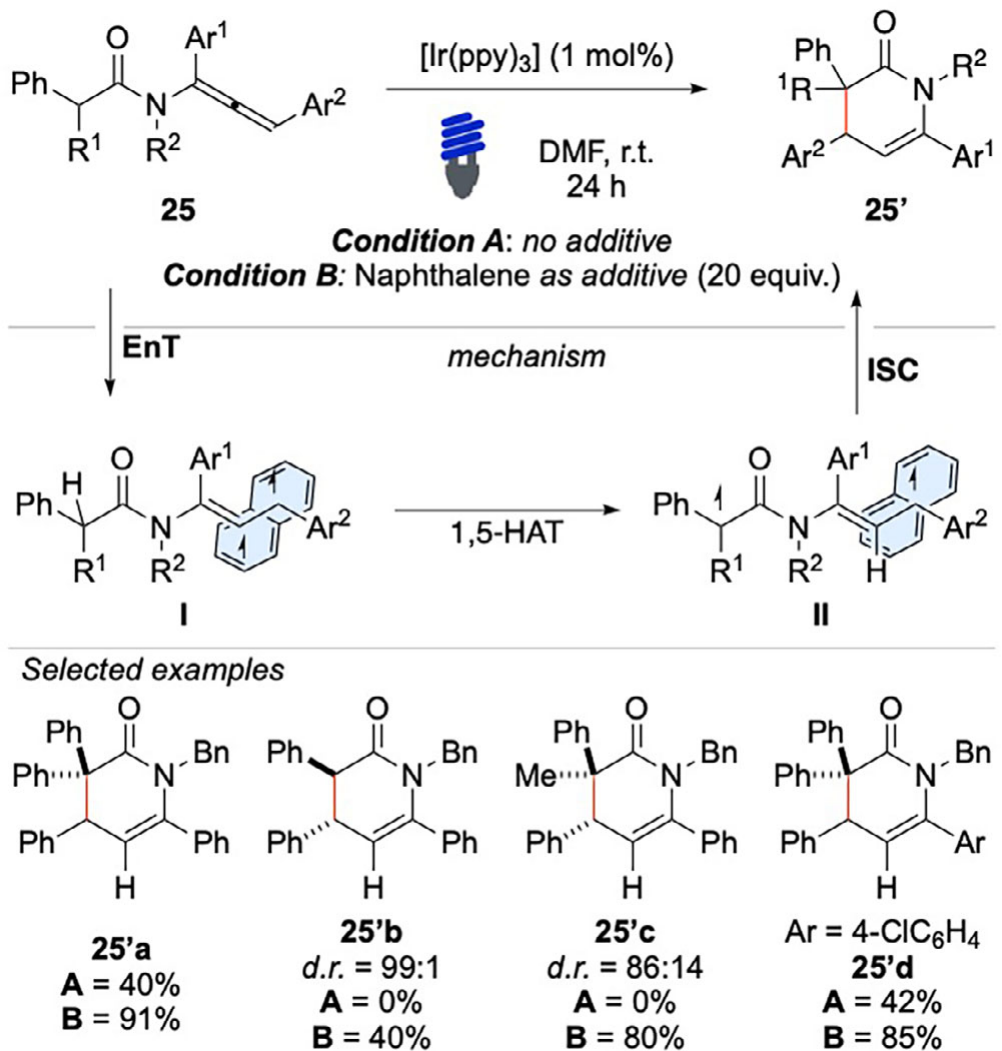
Cascade HAT/cyclization of allenamides **25**.

Recently, our research group has successfully developed a cascade HAT/cyclization of allenamides to form 3,4‐dihydropyridin‐2(1H)‐one **25′** by exploiting dispersion interactions in EnT processes (Scheme 25a).^[^ ^101^ ^]^

The dispersion interactions exerted by naphthalene played a crucial role in enhancing the kinetics of the process, thereby facilitating the otherwise challenging transformation. Through the light‐mediated activation of allene **25**, a triplet with an electrophilic vinyl radical site is generated. The intermediate **I** can abstract a hydrogen atom via selective 1,5‐HAT, providing intermediate **II**, which eventually affords the six‐membered heterocycle **25′**. Notably, the protocol exhibits remarkable tolerance to variations in electronic properties, making it broadly applicable to a diverse range of substrates (Scheme 25b).

Nonetheless, the process requires the use of trisubstituted allenes. Substrates for which the bond dissociation energy (BDE) of the C(sp^3^)─H group undergoing the HAT is less favorable, such as **25′a** and **25′b**, provided the desired product only in the presence of naphthalene. This observation underscores the essential role of this species in the reaction, which, according to DFT modeling data, is due to the generation of stabilizing dispersion interactions between mono‐occupied molecular orbitals of the triplet intermediates and the π‐network of the aromatic compound. The extensive experimental and computational studies conducted to gain insights into these interactions suggested that a further improvement could have been achieved by adopting a multivalent approach. Indeed, by replacing naphthalene with a tethered poly‐naphthyl derivative, it was possible to maintain the positive effect on the reaction kinetics reducing the loading of the additive, from a super‐stoichiometric molar excess (5–20 equiv.), down to a catalytic one (10–30 mol%).^[^ ^102^ ^]^ This effect can be attributed to a reduced entropic cost connected with the formation of the supramolecular adducts between triplets and these co‐catalysts.

**Scheme 26:**
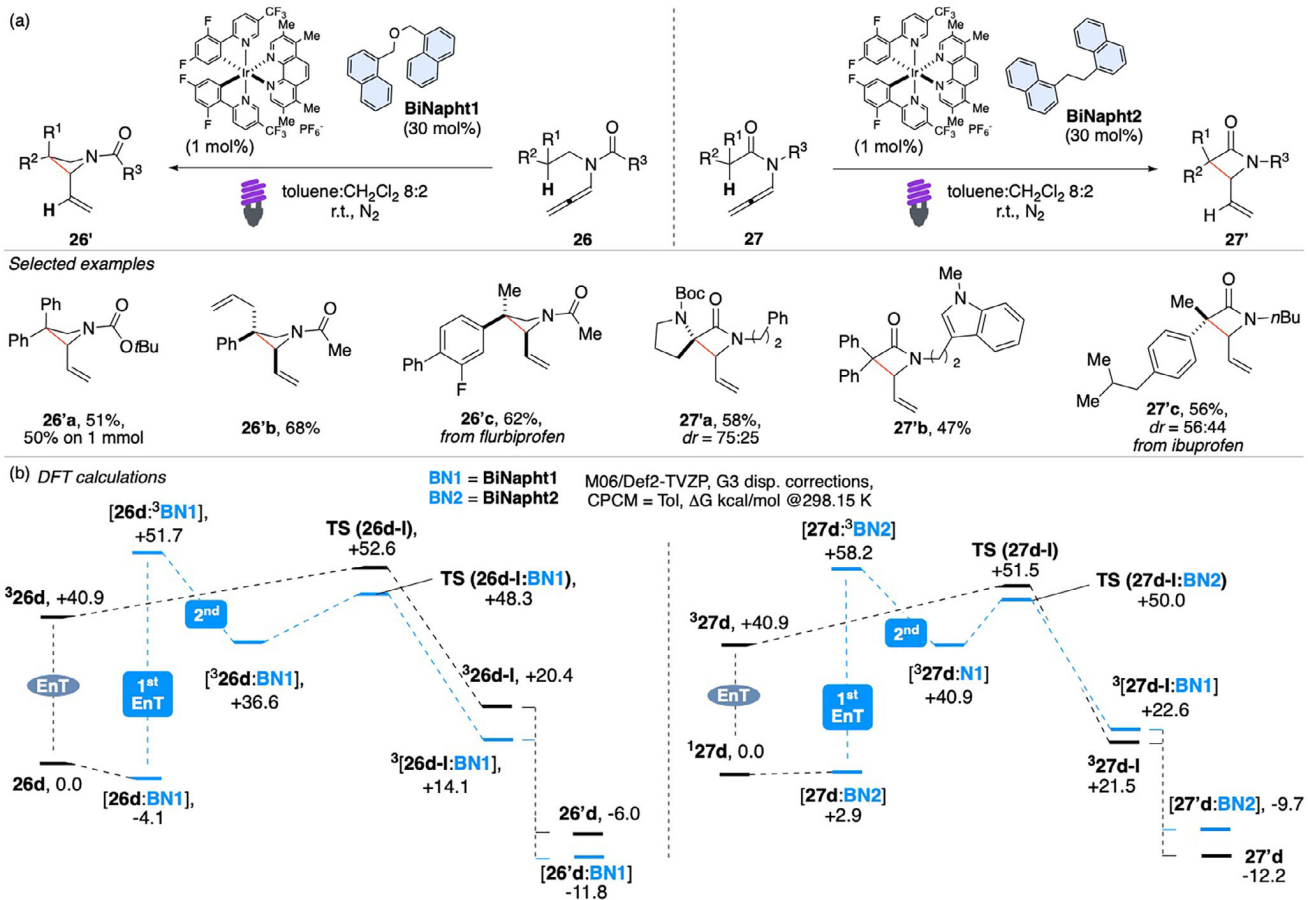
Sequential 1,5‐HAT/cyclization of allenamides **26** and **27** to azetidines **26′** and β‐lactams **27′**.

Azetidines and β‐lactams are important components of natural and pharmaceutical bioactive molecules, however, only a limited number of catalytic methods provide access to densely functionalized nitrogen‐containing four‐membered rings. Building upon our previous success with EnT activation of allenes and the subsequent cyclization cascade involving a 1,5‐HAT, we recently demonstrated that this cascade mechanism is suitable for the synthesis of strained rings such as azetidines and β‐lactams (Scheme 26).^[^ ^103^ ^]^

The catalytic systems employed in this study are composed of an iridium‐based photocatalyst in combination with specifically designed **BiNapht** co‐catalysts. In particular, the use of substrate **26** together with **BiNapht1** facilitated the efficient synthesis of azetidines, while the combination of substrate **27** with **BiNapht2** selectively led to the formation of β‐lactams. These results highlight the crucial role of the **BiNapht** co‐catalysts in steering the reaction outcome toward distinct heterocyclic frameworks, underscoring the modularity and tunability of the catalytic system. The reaction demonstrated an excellent functional group tolerance, effectively accommodating a broad range of structurally and electronically diverse substituents. Notably, it allowed for the successful incorporation of a Boc‐protected amide, as exemplified by the compounds **26′a** and **27′a**. The robustness of the method toward strained rings is further demonstrated by the 1 mmol scale reaction of **26′a**. Additionally, the reaction exhibited compatibility with various aryl and alkyl moieties both on the core of substrates **26** and **27**, including the installation of complex substituents such as pharmaceutical derivatives, as illustrated for **26′c** and **27′c**. The ability to achieve good to excellent yields across this range of substrates is particularly remarkable, especially in light of the inherent complexity and multistep nature of the cascade process involved.

Experiments and DFT calculations elucidate the reaction mechanism and prove the fundamental role of the co‐catalysts **BiNapht**s. Calculated reaction pathways have been reported in Scheme 26b. Initially, **26** and **BiNapht1** can form an adduct, [**26**:**BiNapht1**], thanks to π–π stabilization and C─H─π interactions. Subsequently, two sequential EnTs would follow. The first one is intermolecular and it involves the photoexcited Ir‐photocatalyst and **BiNapht1**, giving [**26**:^3^ **BiNapht1**]. The second EnT is intramolecular and it occurs between ^3^ **BiNapht1** and **26**, affording [^3^ **26**:**BiNapht1**] that has a delocalized allylic mono‐occupied molecular orbital and a reactive vinyl‐radical one. This intermediate can deliver [**26d‐I**:**BiNapht1**], which has a more stable tertiary benzylic radical site, via 1,5‐HAT. The azetidine ring is established upon intersystem crossing via radical recombination, allowing **BiNapht1** to turnover. An analogous manifold affords β‐lactams **27**. The approach features a vinyl radical generated via an atom‐economical HAT step, using a powerful Ir(III) photosensitizer and a **BiNapht** co‐catalyst that stabilizes intermediates. This EnT relay strategy offers a versatile platform for building complex molecular architectures.

**Scheme 27:**
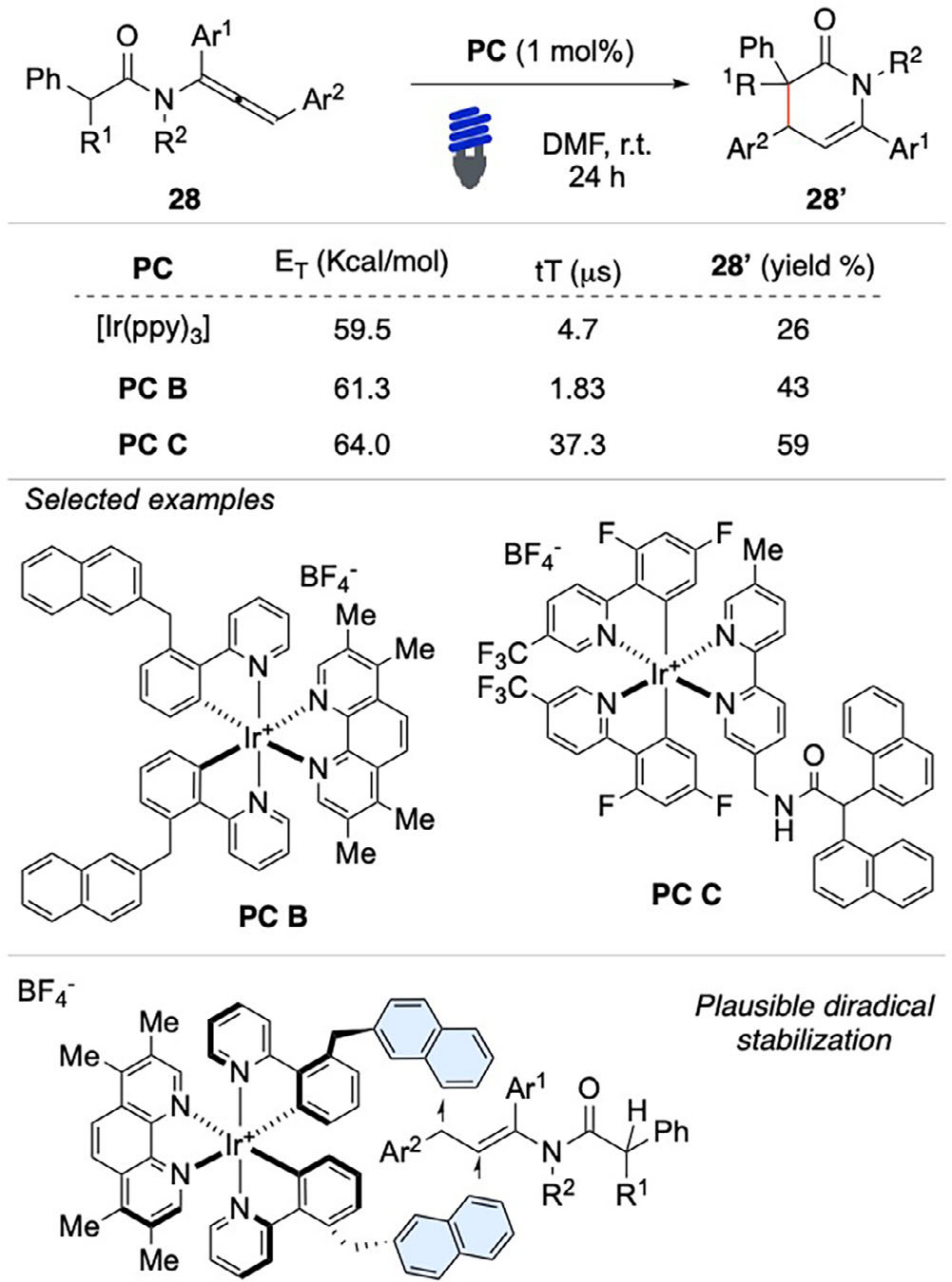
Novel Ir(III) photoactive complexes for EnT processes.

Taking advantage of the efficiency attributed to the (di)radical‐π dispersion interactions between naphthyl rings and transient triplets, our research group has developed a novel class of iridium‐based photosensitizers featuring naphthyl pendant groups (Scheme 27).^[^ ^104^, ^105^ ^]^ By studying the intricate interplay between the architecture of ligands and the resulting photophysical properties of the complexes, we prepared a new family of photocatalysts through the functionalization of phenylpyridines and bipyridines with various (poly)naphthyl units. Among them, the most effective photocatalyst was **C**, which, thanks to its particularly long triplet lifetime and suitable triplet energy, successfully activated substrate **28**, delivering product **28′a** in a reasonable yield, especially when compared to the reaction using commercial [Ir(ppy)_3_], and [Ir(ppy)_3_], which are ineffective for the transformation and become competent species only in the presence of 20 equiv. of naphthalene.

**Scheme 28:**
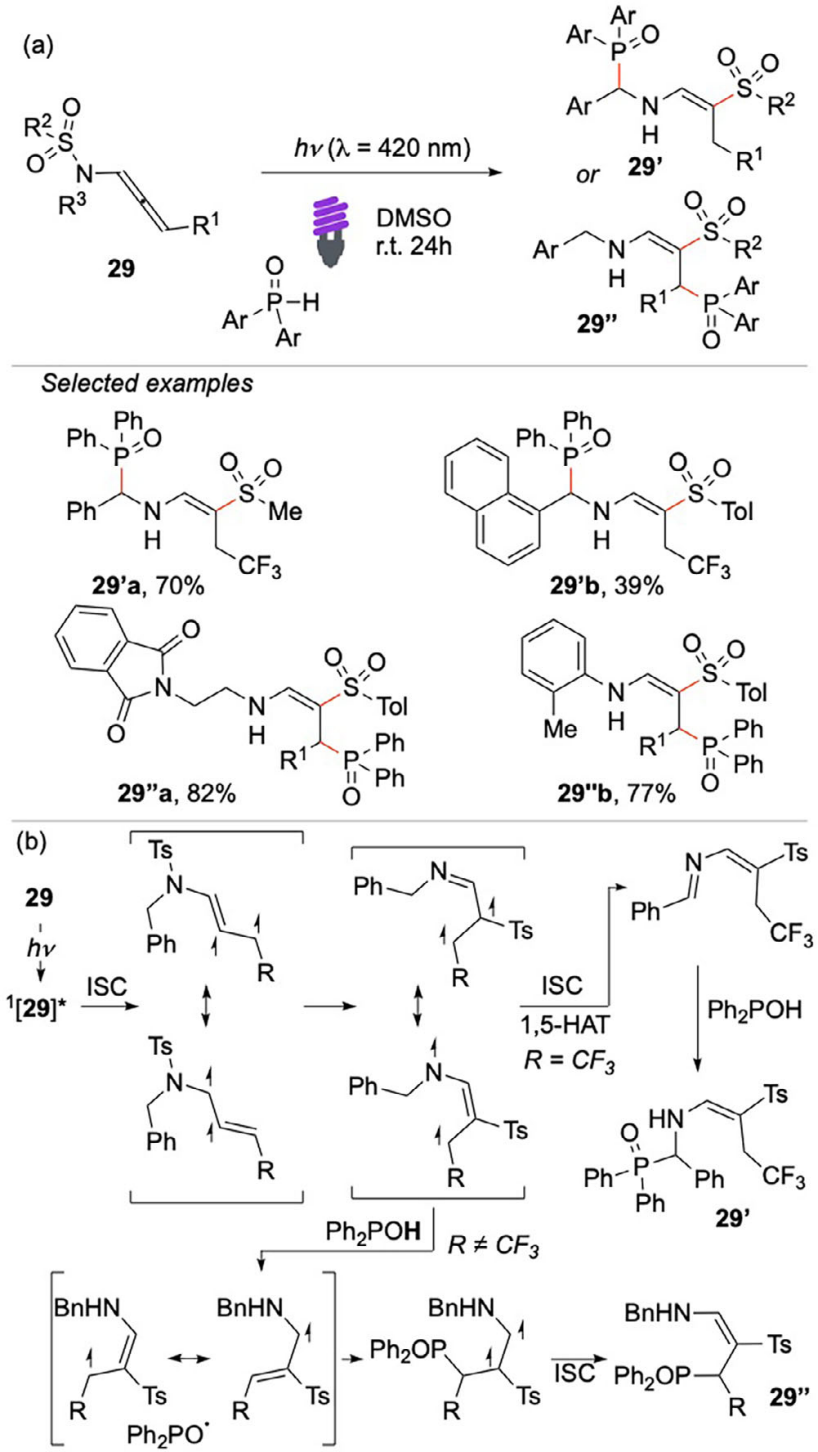
Direct activation of allenamide **29** via visible light.

In 2024, Ciofini and Miesch investigated the reactivity of *N*‐allenamides under visible‐light irradiation.^[^ ^38^ ^]^ In contrast to the previously discussed EnT activation of allenes, they demonstrated a visible‐light–induced direct excitation of the allenamide to its singlet excited state (Scheme 28). Subsequent ISC generates the corresponding triplet state, which governs the observed reactivity. Formation of a diradical vinyl intermediate enables a ^[^ ^1^, ^3^ ^]^‐Ts shift, in analogy analogous to reactivities observed with enallenes, ^[^ ^96^ ^]^ leading to an N‐centered radical. A second ISC event, followed by a 1,5‐HAT, produces the imino‐vinyl sulfone, which, upon quenching with an H‐phosphine oxide, affords the amino‐phosphoryl alkene **29′**. Interestingly, the presence of an electron‐withdrawing CF_3_ group on the allene stabilizes the radical intermediate and induces a switch in regioselectivity, directing the reaction from **29′** to **29′’**. The protocol exhibits broad functional group tolerance, and its successful scale‐up as well as feasibility under sunlight irradiation underscore its sustainability and synthetic utility.

**Scheme 29:**
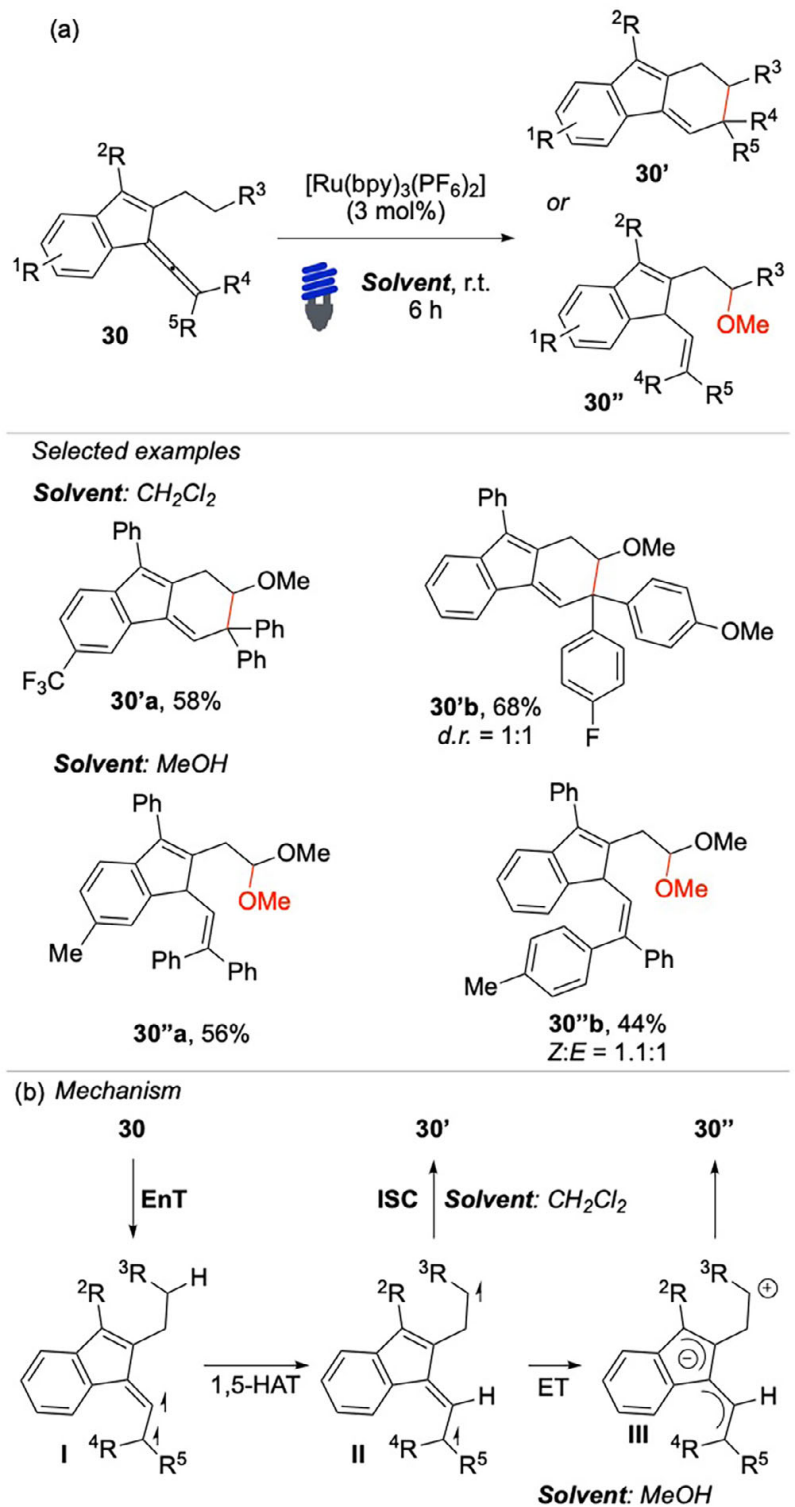
EnT/HAT approach for the activation of C(sp^3^)─H bond of **30**.

In 2021, Shi and colleagues disclosed an innovative approach for the activation of remote C(sp^3^)─H bonds through a consecutive EnT/HAT reaction pathway on aryl‐allenes (Scheme 29).^[^ ^106^ ^]^ The novelty of the procedure lies in the strategic use of the allenyl derivative, which, upon population of its corresponding triplet state, has one of its mono‐occupied molecular orbitals with a vinyl radical character that can trigger a 1,5‐HAT from a C(sp^3^) ─H bond.

Initial studies focused on the cyclization reaction toward 1H‐fluorene **30′**, which was accomplished using the allene **30** as a substrate. Regardless of the substituent of the indenes, the reaction consistently proceeded with remarkable efficiency. The researchers then shifted their attention toward the reactivity of allenes in combination with alcohols (methanol), which involved a radical/polar crossover pathway. These promising results were further substantiated by a robust mechanistic investigation, which incorporated experimental evidence derived from deuterium labeling experiments and kinetic analyses. Additionally, DFT calculations provided valuable insights into the underlying reaction pathways, reinforcing the proposed mechanistic model. The reaction mechanism commences with the excited photocatalyst that activates **30** via EnT, providing triplet intermediate **I** (Scheme 29b). This species undergoes a 1,5‐HAT process, resulting in the formation of intermediate **II**, which has an allylic radical character.

This biradical intermediate can subsequently undergo intramolecular ring‐closure thanks to the ability its allylic double bond to rotate, thereby yielding the final product **30′**. When the reaction is carried out in a highly polar solvent, such as methanol, it is proposed that the biradical intermediate **II** could undergo an intramolecular electron transfer that generates the corresponding zwitterionic intermediate **III**. The latter provides the product **30′’** via sequential nucleophilic attack/1,5‐proton shift.

**Scheme 30:**
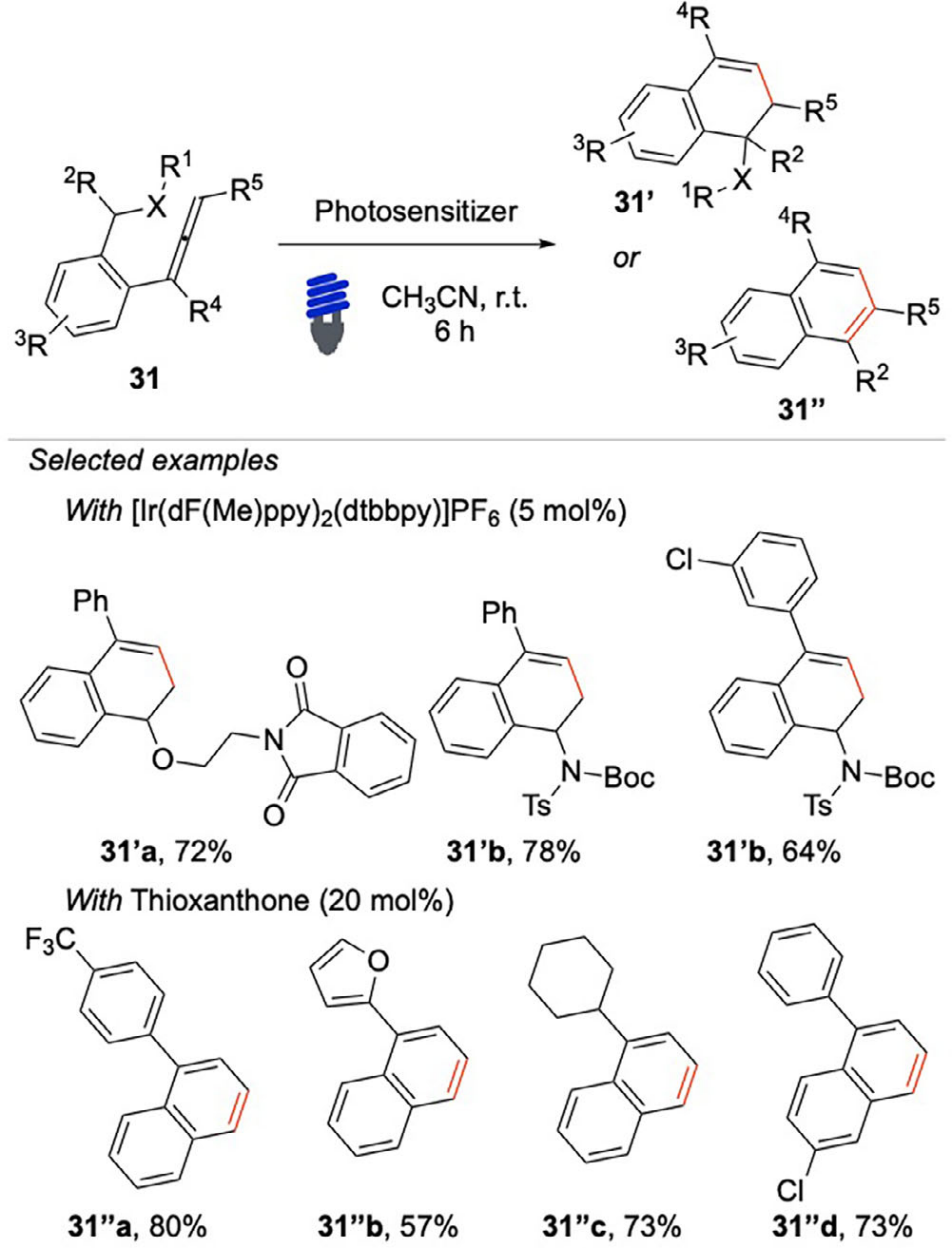
EnT/HAT activation approach of C(sp^3^)─H bond of **31**.

More recently, Liu, Wei, Shi, and coworkers reported a 1,5‐HAT and C(sp^3^)─H bond activation via EnT (Scheme 30).^[^ ^107^ ^]^ Similar to the previous approach, the reaction begins with the EnT‐mediated activation of the aryl‐allene, followed by an intermolecular 1,5‐HAT that generates a stabilized benzylic radical.

This intermediate then undergoes 6π electrocyclization to form a new C─C bond. The strategy operates under mild conditions and demonstrates broad substrate scope, with good functional group tolerance and yields. Interestingly, by judicious selection of the photocatalyst, the chemoselectivity can be tuned to favor the formation of the **31′'** class of products.

**Scheme 31:**
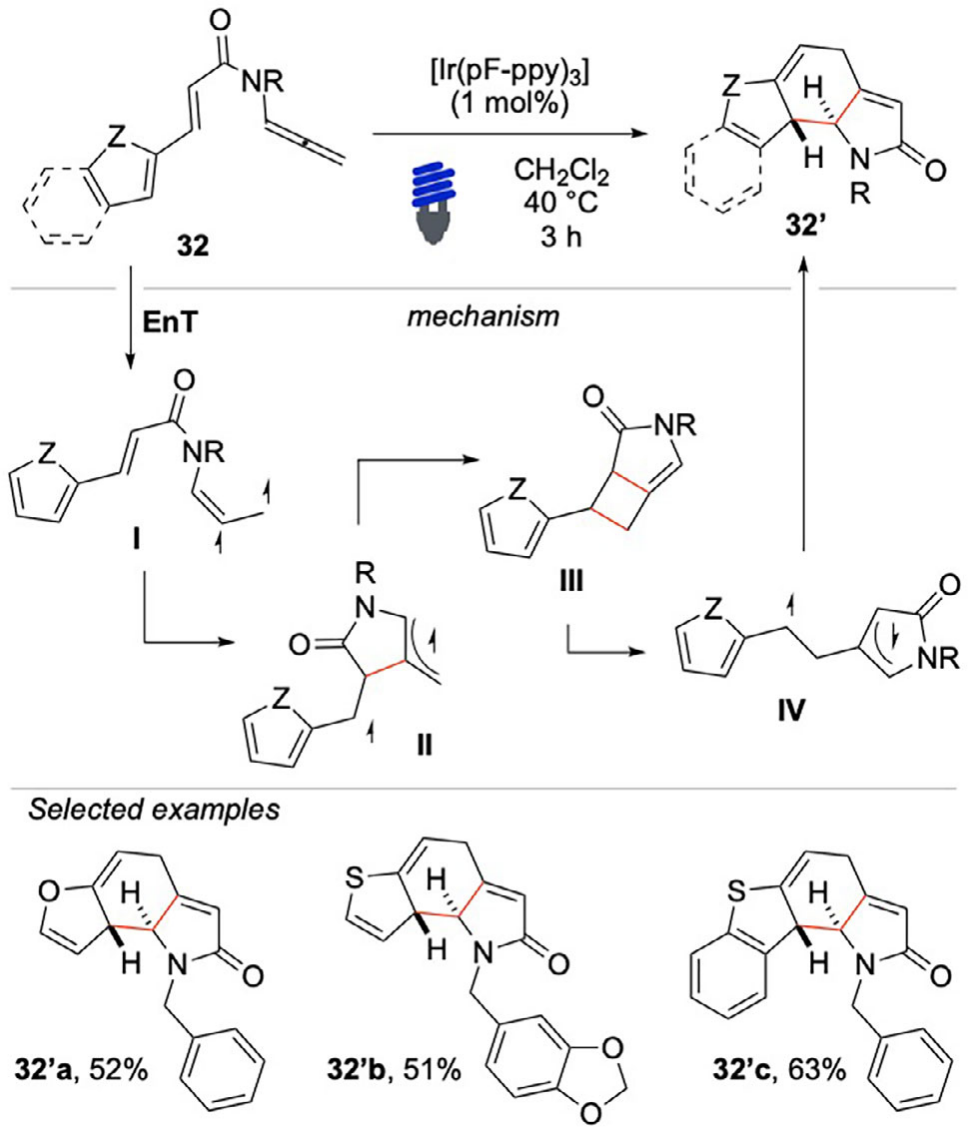
Dearomative skeletal rearrangement via EnT activation of N‐allenamides **32**.

Interested in developing innovative cascade reactions, our group has designed a consecutive dearomative rearrangement of heteroaryl substrates via EnT activation of allenamides, leading to complex fused tricycles (Scheme 31a).^[^ ^108^ ^]^ The key innovation of this approach lies in the unique skeletal rearrangement of the enallene unit. The EnT activation of the substrate provides intermediate **I**. This species undergoes cyclization to generate the lactam intermediate **II**. A subsequent radical recombination yields the strained bicyclic intermediate **III**. Ring‐opening of the vinylidene cyclobutene unit is accompanied by the formation of a more stable biradical intermediate (**IV**), which has an allylic and a benzylic radical character. This species then undergoes isomerization and intramolecular recombination, ultimately producing the final product **32′**. The study demonstrated the robustness of this method by successfully applying it to a variety of heterocycles (Scheme 31b), highlighting its great potential for future advancements, tackling even more challenging dearomative transformations.

**Scheme 32:**
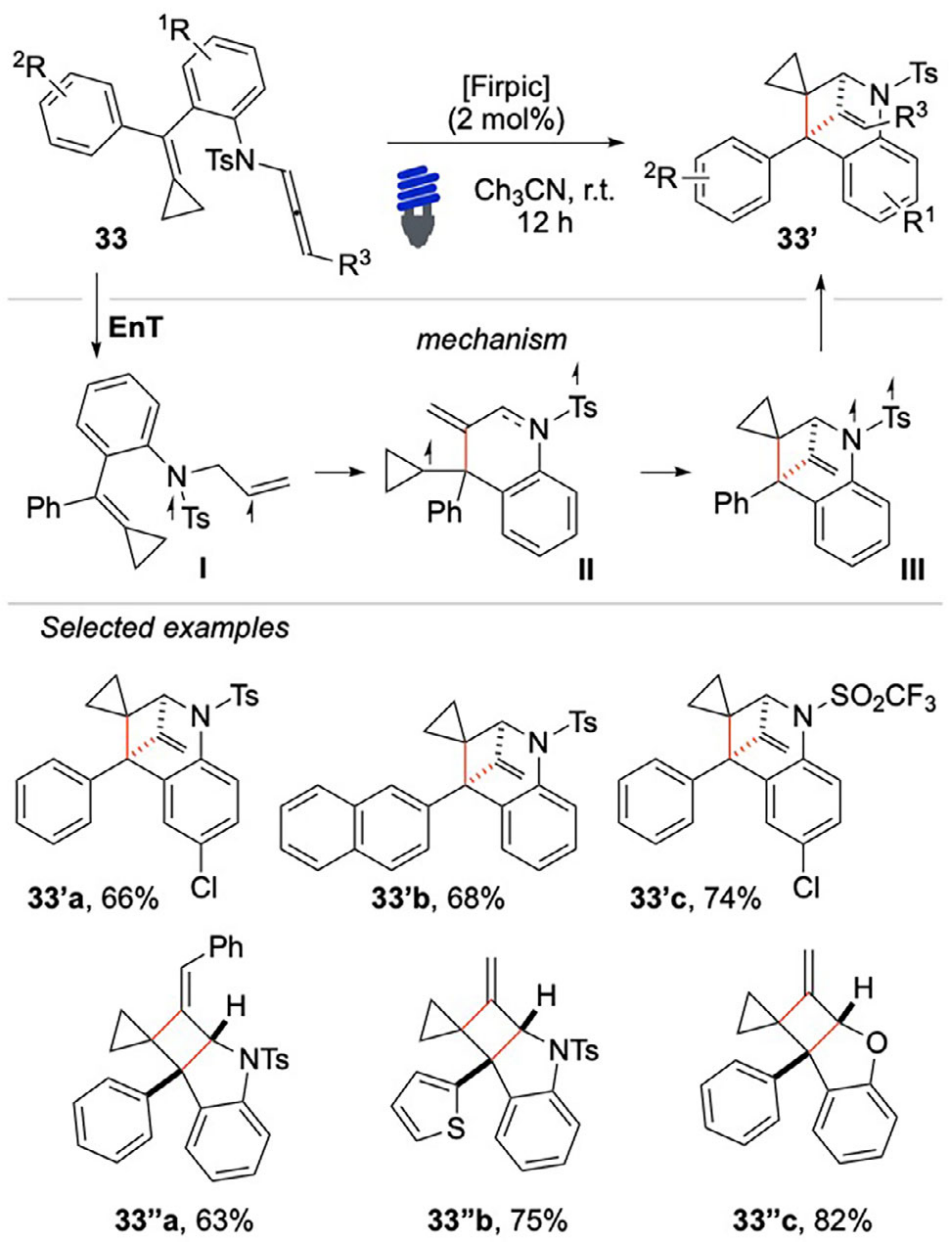
EnT based Intramolecular crossed [2 + 2] cycloaddition.

Recently, Wei, Shi, and coworkers have reported an interesting intramolecular crossed [2 + 2] cycloaddition to access bicyclo[2.1.1]hexanes (Scheme 32a).^[^ ^109^ ^]^ In this study, the EnT activation of the sulfonyl N‐allenamide **30** is achieved by the highly energetic Firpic photosensitizer (*E*_T_ = 60.5 kcal/mol). The authors propose that a peculiar sulfonyl radical character of the triplet intermediates of the reaction is crucial to assist the crossed [2 + 2] cycloaddition. The bridged spirocyclic compounds **33′** was accessible even in the presence of a large variety of functional groups, thus demonstrating the robustness of the methodology (Scheme 32b). The method is a valuable contribution because it is the first case of visible‐light‐promoted crossed [2 + 2] involving the activation of an allene. Nonetheless, some structural limitations were present, such as the requirement to have a tetrasubstituted alkenyl partner decorated by two aryl substituents and a cyclopropyl ring. Although the primary aim of the investigation was the formation of the crossed product **33′**, the [2 + 2] product **33′’** was also often obtained.

EnT photocatalysis has proven highly effective in enabling stereoselective transformations of allenes, including deracemizations, radical cascades, and dearomatizations, granting access to complex, stereodefined architectures. Key challenges remain, particularly in enantioselective radical cascades and dearomatization strategies, as well as in the integration of these processes with flow chemistry. Continued exploration of these approaches promises to fully harness the unique reactivity of allenes, solidifying their role as versatile building blocks in modern synthetic chemistry.

## 4 Conclusions

The reactivity of conjugated dienes is strongly influenced by the diene conformation and the triplet energy of the sensitizer; as a result, photocatalytic EnT sequences can lead selectively to either cis/trans isomerizations or [2 + 2] and [4 + 2] cycloadditions. Advances in the design of the photocatalyst, such as ruthenium and iridium complexes, riboflavin, and quantum dots, allow high chemo‐, regio‐, and stereoselectivity, even in complex natural product synthesis. Recent developments in solar‐driven and mild photocatalytic protocols highlight sustainable and efficient strategies, while challenges remain in scalability and expanding substrate scope. Overall, EnT‐mediated photochemistry provides a versatile tool for stereocontrolled and structurally complex molecule construction.

Allenes are unique organic molecules with cumulated π‐systems, linear central carbons, and axial chirality, giving them distinctive reactivity and stereoelectronic properties. These features make them versatile intermediates for cycloadditions, radical cascades, and photochemical transformations. Recent advances in visible‐light and EnT photocatalysis enable stereoselective deracemizations, dearomatizations, and the construction of complex, sp^3^‐rich architectures. Through precise control of radicals, hydrogen bonding, and dispersion interactions, allenes serve as powerful building blocks for synthesizing complex, functionalized molecules.

In conclusion, these studies highlighted the versatility of EnT‐mediated strategies for the synthesis of complex molecules. The visible‐light‐mediated activation of either 1,3‐dienes or allenes provides a powerful platform for the selective construction of elaborate molecular architectures that would be otherwise difficult to access. The ability to fine‐tune reaction pathways through subtle electronic modifications expands the potential of photochemical methodologies, offering new synthetic opportunities. Moreover, recent contributions from different research groups showed the potential impact of the EnT‐activation of cumulated double bonds, which remains an overlooked synthetic avenue. Therefore, we anticipate vast future opportunities to expand this promising endeavor.

## Conflict of Interest

The authors declare no conflict of interest.

## Data Availability

Data sharing is not applicable to this article as no new data were created or analyzed in this study.
